# Supplementary material for: Key Components, Current Practice and Clinical Outcomes of ERAS Programs in Patients Undergoing Orthopedic Surgery: A Systematic Review
Source: J Clin Med. 2022 Jul 20;11(14):4222. doi: 10.3390/jcm11144222 (PMC9322698; doi:10.3390/jcm11144222)
Supplement: Supplementary file 1 [file jcm-11-04222-s001.zip › jcm-1731803-supplementary.pdf]

Table S1: Search terms used in the PubMed, Scopus, and Web of Science Core Collection.

Database	Free-vocabulary and/or Medical Subject Headings (MeSH) terms
PubMed	(("musculoskeletal diseases"[MeSH Terms] OR ("musculoskeletal"[All Fields] AND "diseases"[All Fields]) OR "musculoskeletal diseases"[All Fields] OR ("orthopedic"[All Fields] AND "disorders"[All Fields]) OR "orthopedic disorders"[All Fields] OR ("orthopaedic surgery"[All Fields] OR "orthopedics"[MeSH Terms] OR "orthopedics"[All Fields] OR ("orthopedic"[All Fields] AND "surgery"[All Fields]) OR "orthopedic surgery"[All Fields])) AND ("fast-track"[All Fields] OR ("enhanced recovery after surgery"[MeSH Terms] OR ("enhanced"[All Fields] AND "recovery"[All Fields] AND "after"[All Fields] AND "surgery"[All Fields]) OR "enhanced recovery after surgery"[All Fields]) OR (("enhance"[All Fields] OR "enhanced"[All Fields] OR "enhancement"[All Fields] OR "enhancements"[All Fields] OR "enhancer"[All Fields] OR "enhancer s"[All Fields] OR "enhancers"[All Fields] OR "enhances"[All Fields] OR "enhancing"[All Fields]) AND ("recoveries"[All Fields] OR "recovery"[All Fields]) AND ("program"[All Fields] OR "program s"[All Fields] OR "programe"[All Fields] OR "programed"[All Fields] OR "programes"[All Fields] OR "programing"[All Fields] OR "programmability"[All Fields] OR "programmable"[All Fields] OR "programmably"[All Fields] OR "programme"[All Fields] OR "programme s"[All Fields] OR "programmed"[All Fields] OR "programmer"[All Fields] OR "programmer s"[All Fields] OR "programmers"[All Fields] OR "programmes"[All Fields] OR "programming"[All Fields] OR "programmings"[All Fields] OR "programs"[All Fields])))) AND (y_10[Filter])
Web of Science Core Collection	(TS = orthopedic disorders OR TS = orthopedic surgery) AND (TS = fast-track OR TS = enhanced recovery after surgery OR TS = enhanced recovery programs) - with Publication Year from 2011 to 2021
Scopus	(TITLE-ABS-KEY (orthopedic AND disorders) OR TITLE-ABS-KEY (orthopedic AND surgery) AND TITLE-ABS-KEY (fast-track) OR TITLE-ABS-KEY (enhanced AND recovery AND after AND surgery) OR TITLE-ABS-KEY (enhanced AND recovery AND programs)) AND PUBYEAR > 2010

Table S2: National Heart, Lung, and Blood Institute (NHLBI) quality assessment tool.

Reference	Criteria													
	1	2	3	4	5	6	7	8	9	10	11	12	13	14
Aasvang et al. 2016 [25]								NA						
Adams et al. 2021 [26]								NA						
Adeyemo et al. 2021 [27]								NA						
Alvis et al. 2021 [28]								NA						
Andreasen et al. 2017 [29]								NA						
Ascione et al. 2020 [30]								NA						
Auyong et al. 2015 [31]								NA						
Awada et al. 2019 [32]								NA						
Bandholm et al. 2014 [33]								NA		NR				
Berg et al. 2018 [34]								NA						
Berg et al. 2020 [35]								NA						
Berg et al. 2021 [36]								NA		NR				
Bernaus et al. 2021 [37]								NA						
Berthelsen et al. 2017 [38]								NA						
Birznieks et al. 2019 [39]							NR	NA		NR				
Bjerregaard et al. 2015 [40]							NR	NA		NR				
Bjerregaard et al. 2016 [41]								NA						

Cao et al. 2020 [42]								NA						
Castle et al. 2021 [43]							NR	NA		NR				
Castorina et al. 2018 [44]								NA						
Christelis et al. 2015 [45]								NA						
Concina et al. 2019 [46]							NR	NA						
Collett et al. 2021 [47]								NA		NR				
Cui et al. 2019 [48]								NA						
D’Amato et al. 2019 [49]								NA						
d’Astorg et al. 2020 [50]								NA						
Dagal et al. 2019 [51]								NA						
Davies et al. 2018 [52]								NA						
Dawson-Bowling et al. 2014 [53]								NA						
De Ladoucette et al. 2020 [54]								NA						
Debono et al. 2019 [55]								NA						
Debono et al. 2021 [56]								NA						
Deiter et al. 2020 [57]								NA						
den Hartog et al. 2015 [58]							NR	NA						
den Hartog et al. 2017 [59]							NR	NA						
Didden et al. 2019 [60]							NR	NA						
Ding et al. 2020 [61]								NA						

Drosos et al. 2016 [62]								NA						
Drosos et al. 2020 [63]								NA						
Dwyer et al. 2012 [64]								NA						
Dwyer et al. 2014 [65]								NA						
Fenelon et al. 2018 [66]							NR	NA						
Feng et al. 2019 [67]								NA						
Fletcher et al. 2020 [68]								NA						
Fletcher et al. 2021 [69]								NA						
Fransen et al. 2018 [70]								NA						
Frassanito et al. 2020 [71]								NA						
Füssenich et al. 2020 [72]								NA						
Galbraith et al. 2017 [73]								NA						
Glassou et al. 2014 [74]								NA						
Gomez et al. 2019 [75]								NA						
Gomez et al. 2020 [76]														
Götz et al. 2021 [77]								NA						
Gromov et al. 2015 [78]							NR	NA						
Gromov et al. 2019 [79]								NA						
Gromov et al. 2020 [80]								NA						
Gupta et al. 2014 [81]							NR	NA						

Halawi et al. 2019 [82]			NR		NR			NA		NR				
Hansson et al. 2015 [83]								NA		NR				
Hartog et al. 2015 [84]								NA						
He et al. 2020 [85]							CD	NA					NR	
Heo et al. 2019 [86]								NA						
Herndon et al. 2020 [87]								NA		NR				
Higgins et al. 2020 [88]								NA		NR				
Holm et al. 2014 [89]								NA		NR				
Holmes et al. 2019 [90]							CD	NA						
Hoorntje et al. 2017 [91]								NA						
Huang et al. 2021 [92]								NA		NR				
Husted et al. 2011a [93]								NA						
Husted et al. 2011b [12]								NA						
Husted et al. 2012 [94]								NA		NR				
Husted et al. 2016 [95]								NA		NR				
Imbelloni et al. 2014 [96]								NA						
Jenny et al. 2020 [97]								NA		NR				
Jensen et al. 2020 [98]								NA		NR				
Jensen et al. 2021 [99]								NA		NR				
Jiang et al. 2019 [100]								NA		NR				

Jørgensen et al. 2013a [24]								NA		NR				
Jørgensen et al. 2013b [101]								NA						
Jørgensen et al. 2017 [102]								NA						
Jørgensen et al. 2021 [103]								NA						
Julien-Marsollier et al. 2020 [104]								NA						
Kang et al. 2019 [105]								NA		NR				
Kerr et al. 2017 [106]								NA		NR				
Kilic et al. 2020 [107]								NA						
Klapwijk et al. 2017 [108]								NA						
Klement et al. 2019 [109]								NA						
Kolodziej et al. 2020 [110]								NA		NR				
Kort et al. 2018 [111]								NA		NR				
Krenk et al. 2012 [112]								NA		NR				
Krenk et al. 2014 [113]								NA						
Lamplot et al. 2014 [114]								NA		NR				
Larsen et al. 2012 [115]								NA						
Larsson et al. 2016 [116]								NA		NR				
Leiss et al. 2021 [117]								NA		NR				
Li et al. 2018 []								NA						
Li et al. 2020a [119]								NA						

Li et al. 2020b [120]								NA		NR				
Li et al. 2021a [121]								NA						
Li et al. 2021b [122]								NA						
Lindberg-Larsen et al. 2017 [123]								NA						
Lindberg-Larsen et al. 2018a [124]								NA						
Lindberg-Larsen et al. 2018b [125]								NA						
Lindberg-Larsen et al. 2019 [126]								NA						
Lovecchio et al. 2016 [127]								NA		NR				
Machin et al. 2013 [128]								NA		NR				
Maempel et al. 2015 [129]								NA						
Mangat et al. 2020 [130]								NA						
McDonald et al. 2012 [131]								NA						
Memtsoudis et al. 2020 [132]								NA						
Mikkelsen et al. 2014 [133]								NA						
Munk et al. 2012 [134]								NA						
Nazarenko et al. 2016 [135]								NA						
Nicolaiciuc et al. 2019 [136]								NA						
Noel et al. 2020 [137]								NA						
Okamoto et al. 2016 [138]								NA		NR				
Otte et al. 2011 [139]								NA		NR				

Pamilo et al. 2018 [140]								NA						
Petersen et al. 2017 [141]							CD	NA						
Petersen et al. 2019 [142]								NA		NR				
Petersen et al. 2020a [143]								NA						
Petersen et al. 2020b [144]								NA						
Petersen et al. 2021 [145]								NA						
Pirsaharkhiz et al. 2020 [146]							CD	NA						
Pitter et al. 2016 [147]								NA						
Plenge et al. 2020 [148]								NA						
Plessl et al 2020 []								NA						
Pollmann et al. 2016 [150]								NA						
Porsius et al. 2018 [151]								NA						
PrkiĆ et al. 2020 [152]								NA						
Robinson et al. 2014 [153]								NA						
Romano et al. 2021 [154]								NA						
Ruiz et al. 2018 [155]								NA						
Rytter et al. 2017 [156]								NA						
Saku et al. 2019 [157]								NA						
Savaridas et al. 2013 [158]								NA						
Schotanus et al. 2017 [159]								NA						

Shaw et al. 2021 [160]								NA						
Skovgaard et al. 2013 [161]								NA						
Soffin et al. 2019b [162]								NA						
Soffin et al. 2019a [20]								NA						
Soffin et al. 2019c [163]							CD	NA						
Soffin et al. 2020 [164]								NA						
Specht et al. 2011 [165]								NA						
Staartjes et al. 2019 [166]								NA						
Stambough et al. 2019 []								NA						
Starks et al. 2014 [168]								NA						
Stowers et al. 2016 [169]								NA						
Talboy et al. 2016 [170]								NA						
Tan et al. 2018 []								NA						
Temporiti et al 2020 [172]								NA						
Tucker et al. 2016 [173]								NA						
van den Belt et al. 2015 [174]								NA						
Van Egmond et al. 2015 [175]								NA						
Van Horne et al. 2019a [176]								NA						
Van Horne et al. 2019b [177]								NA						

Venkata et al. 2018 [178]								NA						
Vesterby et al. 2017 [179]								NA						
Wang et al. 2018a [21]								NA						
Wang et al. 2018b [22]								NA						
Wang et al. 2019 [23]								NA						
Wang et al. 2020 [180]								NA						
Wharton et al. 2020 [181]								NA						
Wied et al. 2015 [182]								NA						
Winther et al. 2015 [183]								NA						
Wynell-Mayow et al. 2018 [184]								NA						
Xie et al. 2019 [185]								NA						
Xu et al. 2019 [186]								NA						
Yang et al. 2020 [187]								NA						
Yanik et al. 2018 [188]								NA						
Yu et al. 2018 [189]								NA						
Zhang et al. 2020 []								NA						
Zietek et al. 2015 [191]								NA						
Zietek et al. 2016 [192]								NA						

1. Was the research question or objective in this paper clearly stated? **2.** Was the study population clearly specified and defined? **3.** Was the participation rate of eligible persons at least 50%? **4.** Were all the subjects selected or recruited from the same or similar populations (including the same time period)? Were inclusion and exclusion criteria for being in

the study prespecified and applied uniformly to all participants? **5.** Was a sample size justification, power description, or variance and effect estimates provided? **6.** For the analyses in this paper, were the exposure(s) of interest measured prior to the outcome(s) being measured? **7.** Was the timeframe sufficient so that one could reasonably expect to see an association between exposure and outcome if it existed? **8.** For exposures that can vary in amount or level, did the study examine different levels of the exposure as related to the outcome (e.g., categories of exposure, or exposure measured as continuous variable)? **9.** Were the exposure measures (independent variables) clearly defined, valid, reliable, and implemented consistently across all study participants? **10.** Was the exposure(s) assessed more than once over time? **11.** Were the outcome measures (dependent variables) clearly defined, valid, reliable, and implemented consistently across all study participants? **12.** Were the outcome assessors blinded to the exposure status of participants? **13.** Was loss to follow-up after baseline 20% or less? **14.** Were key potential confounding variables measured and adjusted statistically for their impact on the relationship between exposure(s) and outcome(s)? **Yes** **No**. **CD**, cannot determine; **NA**, not applicable; **NR**, not reported.

Table S3. Basic characteristics of included literatures studies on spine orthopedic surgery.

**References**	**Study design**	**Pathological condition**	**Patients number, age (years) and gender (%)**	**Surgical procedure**	**Follow-up**	**Outcomes/Endpoints**
Adeyemo et al. 2021 [27]	Retrospective	Degenerative scoliosis	124 patients:<br><br>-Fast-track group (n=67, mean age 68.49±8.72, 60% females);<br><br>-Non-fast-track group (n=57, mean age 69.7±8.23, 67% females)	Thoraco-lumbar-pelvic fusion	90 days	↓Opioid consumption (248,05 mg *vs.* 314,05 mg), urinary retention requiring catheterization (5,97% *vs.* 19,3%), severe constipation (1,49% *vs.* 31,57%), readmissions (2,98% *vs.* 28,07%), blood loss intraoperatively (1284,84 ml *vs.* 1691.8 ml) in fast-track group *vs.* non-fast-track group
Dagal et al. 2019 [51]	Retrospective	NR	450 patients:<br><br>-Fast-track group (n=267, mean age 60±12, 55.4% females);	Major elective spine surgery	30 days	↓LOS, ILOS, costs and ICU admissions (48% *vs.* 60%) in fast-track *vs.* non-fast-track group

			-Non-fast-track group (n=183, mean age 61±14, 56.2% females)			
d’Astorg et al. 2020 [50]	Retrospective	Spinal deformities	386 patients:<br><br>-Fast-track group (n=193, mean age 46±12);<br><br>-Non-fast-track group (n=193, mean age 46±13)	Microdiscectomy, arthrodesis, stenosis	1 year (arthrodesis) and 3 months (microdiscectomy)	↓LOS in fast-track group *vs.* non-fast-track group (2,6 *vs.* 4,4 days)
Debono et al. 2019 [55]	Retrospective	Scoliosis and large deformities	3483 patients:<br><br>-Fast-track group (n=1920: 202 ALIF, mean age 46.3±10.7, 49% females; 612 ACDF, mean age 48.7±8.7, 49% females; 1106 posterior fusion, mean age 56.1±10.2, 50.9% females);<br><br>-Non-fast-track group (n=1563: 159 ALIF, mean age 44.5±8.6, 56.6% females; 749 ACDF, mean age 47.6±9.9, 45.6 females; 655 posterior fusion, mean age	ALIF, ACDF, posterior lumbar fusion	90 days	↓LOS in fast-track group *vs.* non-fast-track group (3.33±0.8 *vs.* 6.06±1.1 days for ALIF, 1.3±0.7 *vs.* 3.08±0.9 for ACDF, 4.8±2.3 *vs.* 6.7±4.8 for posterior fusion). ↓complications for lumbar fusions in fast-track group (10.9% *vs.* 14.8%)

			53.8±14.3, 49.7% females)			
Debono et al. 2021 [56]	Retrospective	Radiculopathy with disc prolapse either hard (osteophytic) or soft and failed conservative treatment	404 patients:<br>-Fast-track group (n=202, mean age 48.5±10.6, 49% females);<br>-Non-fast-track group (n=202, mean age 48.7±9.2, 47.5% females)	ACDF	30 and 90 days, 12 months	↓LOS in fast-track group *vs.* non-fast-track group (1.40±0.6 *vs.* 2.96±1.35 days)
Feng et al. 2019 [67]	Retrospective	Lumbar spinal stenosis, spondylolisthesis, degenerative lumbosacral spine diseases, radiculopathy, or neurogenic claudication	74 patients:<br>-Fast-track group (n=44, mean age 61±10, 63.6% females);<br>-Non-fast-track group (n=30, mean age 59±9, 70% females)	MIS-TLIF	30 days	↓LOS (5 *vs.* 7 days), costs, blood loss, operative time, fluid infusion and drainage in fast-track group *vs.* non-fast-track group
Fletcher et al. 2020 [68]	Retrospective	NMS	197 patients (13.2±3.2 age, 110 females, 87 males):<br>-Fast-track + LOS <3 days group (n=56);<br>-Fast-track + LOS 3-7 days group (n=1111);	PSF	180 days	↓Operative time, levels fused, fusion to pelvis, pulmonary complication, blood transfusion in LOS <3 days group *vs.* LOS 3-7 days and LOS >7 days groups

			-Fast-track + LOS >7 days group (n=30)			
Fletcher et al. 2021 [69]	Prospective	AIS	276 patients:<br><br>-Fast-track group (n=203, mean age 14.3±2.1, 78.8% females);<br><br>-Non-fast-track group (n=73, mean age 16.09±2.1, 80.2% females)	PSF	4 weeks	↓LOS (2,2 *vs.* 4,8 days), operative time (2,8 *vs.* 4,8 h), blood loss (240 *vs.* 500cc), curve magnitudes (54,0° *vs.* 62,0°), osteotomies (46% *vs.* 94%), levels fused (10,1±2,6 *vs.* 11,4±1,6) in fast-track group *vs.* non-fast-track group
He et al. 2020 [85]	Prospective	Lumbar disc herniation, stenosis, or spondylolisthesis with unilateral radiculopathy	40 patients:<br><br>-Fast-track + TXA group (n=20, mean age 57.95±12.44, 60% females);<br><br>-Non-fast-track group (n=20, mean age 57.9±11.76, 45% females)	One-level or two-level TLIF surgery	NR	↓Blood loss and ambulation time in fast-track + TXA group *vs.* non-fast-track group
Heo et al. 2019 [86]	Retrospective	Low-grade degenerative spondylolisthesis, low-grade isthmic spondylolisthesis, central stenosis with instability, central	69 patients:<br><br>-Fast-track group (endoscopic TLIF, n=23, mean age 61.4±9.4, 69.6% females);<br><br>-Non-fast-track, (microscopic TLIF, n=46, mean age	Microscopic or endoscopic TLIF	Mean 13.4±2.5 months	↑VAS score (days 1 and 2) in non-fast track *vs.* fast-track group. ↑operative time and ↓EBL in fast-track group *vs.* non-fast-track group. complications=6 in non-fast-track group and 2 in fast-track group

		stenosis with concomitant foraminal stenosis	63.5±10.5, 58.6% females)			
Julien-Marsollier et al. 2020 [104]	Retrospective	AIS	163 patients (<18 age):<br><br>-Fast-track group (n=81);<br><br>-Non-fast-track group (n=82)	PSF	30 days	↓LOS, morphine consumption (25% and 35%, days 2 and 3), constipation (day 2), pain intensity at rest and movement (day 2 and 3) in fast-track group *vs.* non-fast-track group
Kilic et al. 2020 [107]	Retrospective	Idiopathic lumbar scoliosis, degenerative spondylolisthesis, spinal canal stenosis	174 patients:<br><br>-Fast-track group (n=86, mean age 54.79±13.73, 53.4% females);<br><br>-Non-fast-track group (n=88, mean age 49.77±16.96, 53.4% females)	Lumbar spine instrumentations	30 days	↓Intraoperative blood loss, blood transfusion rate, LOS, pain scores, total costs, first oral intake and mobilization in fast-track group *vs.* non-fast-track group
Li et al. 2018 [118]	Retrospective	Degenerative multilevel spine compression, spinal canal stenosis	224 patients:<br><br>-Fast-track group (n=114, mean age 58.53±10.71, 42.1% females);<br><br>-Non-fast-track group (n=110, mean age 56.88±8.82, 39% females)	Cervical laminoplasty	3 days	↓LOS (5.75±2.46 *vs.* 7.67±3.45 days), assisted walking, urinary catheters time, drainage catheters time, VAS score (2.72±0.46 *vs.* 3.35±0.46) maximum VAS score (3.76±1.12 *vs.* 4.35±1.15), food-taking

						in fast-track group *vs.* non-fat-track group
Li et al. 2020a [119]	Retrospective	Lumbar stenosis with instability, scoliosis/ spondylolisthesis	Fast-track group: 260 patients	Open posterior lumbar fusion surgery	30 days	Overall compliance rate: 92.9% (91 patients in the higher compliance group and 169 patients into the lower compliance group). LOS of patients with higher compliance shorter than that of patients with lower compliance. ↓complications in patients with higher compliance group
Li et al. 2021a [121]	Retrospective	Lumbar stenosis	127 patients:<br>-Fast-track group (n=60, mean age 73.6±3.2, 63.3% females);<br>-Non-fast-track (n=67, mean age 74.3±4.2, 59.7% females)	Open lumbar arthrodesis	30 days	↓LOS (13.6±4.0 *vs.* 15.6±3.9 days), complications (8.3% *vs.* 20.9%), VAS (day 1 and 2) in fast-track group *vs.* control group
Nazarenko et al. 2016 [135]	Prospective	Lumbosacral spine herniated intervertebral discs	48 patients:<br>-Fast-track group (n=23, mean age 44.3, 39.1% females);	Microdiscectomy	1, 3 and 6 months	↓VAS pain (by 10%, at discharge and 1 month), LOS (by 39%), and ↑functional activity (Oswestry index and Roland-Morris scale, by

			-Non-fast-track group (n=25, mean age 42.2, 44% females)			20%) in fast-track group *vs.* non-fast-track group
Shaw et al. 2021 [160]	Retrospective	AIS	78 patients:<br><br>-Fast-track + methadone group (n=26, mean age 15.1±1.9);<br><br>-Fast-track group (no methadone, n=52, mean age 14.9±1.9)	PSF	90 days	↓LOS (2.7±0.7 *vs.* 3.1±0.6) in fast-track + methadone group *vs.* fast-track alone group
Soffin et al. 2019b [162]	Retrospective	NR	61 patients:<br><br>-Fast-track + microdiscectomy group (n=34, mean age 46, 50% females);<br><br>-Fast-track + decompression group (n=27, mean age 65, 48.4% females)	Lumbar microdiscectomy or decompression	90 days	↓LOS, operative time (48.8±12.7 *vs.* 64.1±28.6 min) in fast-track + microdiscectomy group *vs.* fast-track + decompression group
Soffin et al. 2019a [20]	Retrospective	NR	33 patients:<br><br>-Fast-track and ACDF group (n=25, mean age 58, 80% females);<br><br>-Fast-track and CDA group (n=8, mean age 44, 75% females)	ACDF or CDA	90 days	=Operative time, EBL, LOS

Soffin et al. 2019c [163]	Retrospective	NR	36 patients:<br>-Fast-track + OFA group (n=18, mean age 1.5±18.92,44.4% females, 10 males);<br>-Fast-track + OCA group (n=18, mean age 60.14±15.4, 44.4% females)	Elective lumbar decompression	NR	↓Perioperative opioid use (2.43±0.86 *vs.* 38.125±6.11 OMEs), LOS in fast-track + OFA group *vs.* fast-track + OCA group
Soffin et al. 2020 [164]	RCT	NR	51 patients:<br>-Fast-track group (n=25, mean age 55±18, 44% females);<br>-Non-fast-track group (n=26, mean age 54±13, 69.2% females)	Primary one- or two-level lumbar fusion	56 days	↑QoR40 scores (day 3) and ↓LOS, oral intake time, patient-controlled analgesia time, opioid use (day 1) and C-reactive protein (day 3) in fast-track group *vs.* non-fast-track group
Staartjes et al. 2019 [166]	Prospective	Lumbar disc herniation, spinal stenosis, spondylolisthesis, facet cysts, or proven DDD	Fast-track group: 2579 patients, mean age 48.5±13.5, 45.9% females	Tubular microdiscectomy, single-level robot-guided PLIF or TLIF, mini-open ALIF, or mini-open decompression	6 weeks, 1 and 2 years	LOS=1.1±1.2 days; 30-day readmissions=20 (0.8%) and 60-day readmissions=36 (1.4%); discharge after 1-night=94% patients. Over the 5-year period: ↑%patients discharge after 1-night, and ↓adverse events. For fusion procedures: ↑1-night hospital stays rate (from 26% to 85%) and ↓LOS (from 2.4±1.2 days

						to 1.5±0.3 days), nursing costs (of 46.8%)
Venkata et al. 2018 [178]	Prospective	Degenerative lumbar and cervical spinal conditions causing neural compression	Fast-track group: 237 patients (mean age 57, 40% females)	Non-instrumented lumbar and cervical spinal decompression and discectomy surgery	1 year	↓LOS: short stay=12 patients (5%), ambulatory=225 (95%) and day surgery after admission=126 (53.2%); readmissions=7 (2.5%)
Wang et al. 2020 [180]	Retrospective	Lumbar disk herniation or spinal stenosis	190 patients:<br><br>-Fast-track group (n=95, mean age 72.39±6.12, 52.6% females);<br><br>-Non-fast-track, (n=95, mean age 70.81±6.27, 57.8% females)	Lumbar fusion surgery	30 days	↓LOS (12.30±3.03 *vs.* 15.50±1.88) in fast-track group *vs.* non-fast-track group
Yang et al. 2020 [187]	Prospective	AIS	Fast-track group: 46 patients (mean age 14.3, 89.1% female)	PSF	15 days	LOS=3.3 days: discharge day 2=1 patient, day 3=33, day 4=9, day 5=3. 80% of patients felt that they were discharged at an appropriate time while 20% felt they were discharged too early. ↑mean FACES pain scores in patients who felt they were discharged early than those who felt

						they were discharged appropriately

Abbreviations: n = number; ↓ = decrease; vs. = versus; NR = not reported; LOS = hospital length of stay; ILOS = intensive care unit length of stay; ICU = intensive care unit; ALIF = anterior lumbar interbody fusion; ACDF = anterior cervical discectomy and fusion; TLIF = transforaminal lumbar interbody fusion; MIS = minimally invasive surgery; NMS = neuromuscolar scoliosis; PSF = Posterior spinal fusion; AIS = adolescent idiopathic scoliosis; h = hours; TXA = tranexamic acid; ↑ = increase; VAS = visual analog scale; EBL = estimated blood loss; CDA = cervical disc arthroplasty; OFA = Opioid-free anesthesia; OCA = opioid-containing anesthesia; OMEs = oral morphine equivalents; RCT = randomized controlled trial; QoR40 = Quality of Recovery 40; DDD = degenerative disc disease;. PLIF = posterior lumbar interbody fusion.

Table S4. Basic characteristics of included literatures studies on thorax orthopedic surgery.

References	Study design	Pathological condition	Patients number, age (years) and gender (%)	Surgical procedure	Follow-up	Outcomes/Endpoints
Holmes et al. 2019 [90]	Retrospective	PE	436 patients:<br><br>-Fast-track group (n=186, mean age 15.3±2.3);<br><br>-Transition group (n=104, mean age 15.5±1.9);<br><br>-Non-fast-track group (n=146, mean age 14.2±3.3)	MIRPE	NR	↓Opioid use (0.5±0.2 *vs.* 0.7±0.4 *vs.* 0.7±0.8 MEDD/kg), LOS (3 *vs.* 4 *vs.* 5 days), first ambulation and catheter removal time in fast-track group *vs.* transition and non-fast-track groups
Mangat et al. 2020 [130]	Retrospective	Pediatric pectus deformity	55 patients:<br><br>-Fast-track group (n=38, mean age 16, 21% females);	Minimally invasive and traditional corrective procedures	30 days	↓Pain score, operative time, opioid use, pruritus in fast-track group *vs.* non-fast-track group

			-Non-fast-track group (n=17, mean age 15, 23.5% females)			
Pirsaharkhiz et al. 2020 [146]	Retrospective	Blunt thoracic trauma resulting in rib fractures	42 patients (mean age 59, 35.5% females)	Urgent or elective thoracic surgical procedure with ESP block	NR	Block effective in 83.3% of patients, no mortality or complications
Wharton et al. 2020 [181]	Retrospective	PE	109 patients:<br><br>-Fast-track group (n=58);<br><br>-Non fast-track group (n=51)	Nuss procedure for surgical correction	3 weeks	↓LOS (2.90 *vs.* 3.49), urinary catheter use and pain scores (day 0) in fast-track group *vs.* non-fast-track group

Abbreviations: PE = pectus excavatum; n = number; MIRPE = minimally invasive repair of pectus excavatum; NR = not reported; ↓ = decrease; vs. = versus; MEDD = Morphine Equivalent Daily Dose; LOS = hospital length of stay; ESP = erector spinae plane.

Table S5. Basic characteristics of included literatures studies on elbow orthopedic surgery.

References	Study design	Pathological condition	Patients number, age (years) and gender (%)	Surgical procedure	Follow-up	Outcomes/Endpoints
Cui et al. 2019 [48]	RCT	Post-traumatic stiff elbow	50 patients:<br><br>-Fast-track group (n=25, mean age 36.68±10.63, 28% females);<br><br>-Non-fast-track group (n=25, mean age	Open arthrolysis	1-5 days, 6 weeks, 6 months	↓VAS pain score (1-5 days), drain time and ↑ROM in fast-track group *vs.* non-fast-track group

			40.88±11.95, 40% females)			
PrkiĆ et al. 2020 [152]	Retrospective	Primary and secondary OA	126 patients:<br>-Fast-track + casting group (n=70, mean age 69±7.8,85.7% females);<br>-Fast-track + non-casting group (n=31, mean age 70±6.6, 80.6% females);<br>-Fast-track + functional discharge group (n=25, mean age 71±8.3, 92% females)	TEA	Mean 45 months	↓LOS (5.4 *vs.* 6.2 days) in non-casting group *vs.* casting group, and (3.8 *vs.* 6.0 days) in functional discharge group *vs.* non-casting group

Abbreviations: RCT = randomized controlled trial; n = number; ↓ = decrease; VAS = visual analog scale; ↑ = increase; ROM = range of motion; vs. = versus; OA = osteoarthritis; TEA = total elbow arthroplasty; LOS = hospital length of stay.

Table S6. Basic characteristics of included literatures studies on hip and/or knee orthopedic surgery.

References	Study design	Pathological condition	Patients number, age (years) and gender (%)	Surgical procedure	Follow-up	Outcomes/Endpoints
Knee						
Aasvang et al. 2016 [25]	Prospective	OA	115 patients:<br>-Fast-track + non-opioid group (n=57,	Unilateral TKA	6 days	↑Postoperative pain (at rest and walk), opioid use in opioid groups *vs.* non-opioid group

			mean age 65, 66% females);<br><br>-Fast-track + low dose opioid group (<30 mg/day morphine equivalents, n=35, mean age 70, 80% females);<br><br>-Fast-track + high dose opioid group (≥30 mg/day morphine equivalents, n=23, mean age 65, 48% females)			
Ascione et al. 2020 [30]	Prospective	NR	481 patients (704 TKA, mean age 69.8, 62.3% females)	TKA	12 months	LOS=2.3 days. 88.5% patients (n=623) satisfied, 2.1% (n=15) dissatisfied. Complications=15 (2%: 5 painful knees, 3 knee stiffness, 3 hematomas, 2 infections, 1 DVT); readmission=1; operative time=40.7 min
Auyong et al. 2015 [31]	Retrospective	NR	252 patients:<br><br>-Fast-track group (n=126, mean age 66.02±10.02, 65% females);<br><br>-Non-fast-track group (n=126, mean age	TKA	30 days	↓LOS (56.1 *vs.* 76.6 h), NRS pain score, opioid use (day 1 and 2), adverse events, need transfusions (21.4% *vs.* 7.9%), and ↑discharge (41% *vs.* 21%), distance ambulated (day 1 and 2) in fast-track

			68.44±9.98, 67.4% females)			group *vs.* non-fast-track group
Bandholm et al. 2014 [33]	Retrospective	NR	15 patients (mean age 72.0±10.1, 73.3% females)	TKA	NR	↑Pain with increasing load, and repetitions to contraction failure
Cao et al. 2020 [42]	RCT	OA	102 patients:<br><br>-Fast-track + full-course tourniquet group (n=51, mean age 64.9±7.5, 72.5% females);<br><br>-Fast-track + second half-course tourniquet group (n=51, mean age 65.2±6.4, 74.5% females)	TKA	2 weeks	↓VAS, thigh circumference growth rate, swelling, TNF-α, PTX3, CCL2, PGE2, SOD-1, Mb, postoperative blood loss, drain volume, blood transfusion, LOS, and ↑ROM, intraoperative blood loss in second half-course group *vs.* full-course group
Castorina et al. 2018 [44]	Retrospective	OA	132 patients:<br><br>-Fast-track group (n=95, mean age 71.10±7.77);<br><br>-Non-fast-track group (n=37, mean age 74.62±6.42)	TKA	3 weeks	↑ROM, MRC, and ↓VAS pain score in fast-track group *vs.* non-fast-track group
Collett et al. 2021 [47]	Retrospective	OA	296 patients:<br><br>-Fast-track group (n=100, mean age 66.7, 4% female);	TKA	Until discharge	↓LOS (66.8 *vs.* 22.3 hours), ↓inpatient opioids (169.5 mg *vs.* 66.7 mg), ↓intraoperative opioids use (57.4 mg *vs.* 10.5 mg), ↓post-anesthesia opioid use (13.6 mg *vs.*

			-Non-fast-track group (n=196, mean age 68.2, 6.1% female)			1.3 mg) in fast-track group *vs.* non-fast-track group
Concina et al. 2019 [46]	Retrospective	NR	151 patients:<br><br>-Fast-track + tourniquet and suction drain (n=51, mean age 73);<br><br>-Fast-track + no tourniquet and no suction drain (n=50, mean age 70);<br><br>-Fast-track + tourniquet (n=50, mean age 75)	Primary TKR	NR	↑Intraoperative blood loss in no tourniquet and no suction drain group *vs.* tourniquet and suction drain and tourniquet groups. ↓Hb in tourniquet and suction drain group *vs.* tourniquet group. ↑transfusion rate, pain and >difficulties in reaching a 90 degrees of knee flexion in tourniquet and suction drain group *vs.* the other groups
Deiter et al. 2020 [57]	Retrospective	OA	150 patients:<br><br>-Fast-track + adductor canal block group (n=75, mean age 67.6±7.8, 49.3% females);<br><br>-Fast-track (n=75, mean age 67.2±7.5, 49.3% females)	Elective TKA	12 and 24 h	↓NRS pain scores (by 90% in PACU and 38% at 12 and 24 h), MME (by 51%) in fast-track + adductor canal block group *vs.* fast-track alone group
Didden et al. 2019 [60]	Retrospective	OA	170 patients:<br><br>-Fast-track group (n=85, mean age 69.0, 52.9% females);	TKA	NR	↑Functional recovery (4 *vs.* 2 days), and ↓LOS (4 *vs.* 7 days), readmissions in new fast-track group *vs.* non-fast-track group

			-Non-fast-track group (n=85, mean age 69.0 age, 57.6% females)			
Drosos et al. 2016 [62]	Prospective	OA	90 patients:<br><br>-Fast-track (n=30, mean age 71.77±6.50, 80% females);<br><br>-Fast-track + IV TXA group (n=30, mean age 69.27±7.21, 80% females);<br><br>-Fast-track + topical TXA group (n=30, 71.10±6.32 age, 80% females)	TKR	1, 2, 4 and 30 days	↓Blood loss (by 16.3% and 21.9%), transfusion rates (by 90% and 93%), number of units in IV TXA and topical TXA groups *vs.* fast-track without TXA group
Dwyer et al. 2014 [65]	Retrospective	NR	112 patients:<br><br>-Fast-track group (n=57, mean age 70, 70.1% females);<br><br>-Non-fast-track group (n=55, mean age 73, 60% females)	TKR	3 weeks	↓LOS (by 29%) in fast-track group *vs.* non-fast-track group
Fransen et al. 2018 [70]	RCT	NR	49 patients:<br><br>-Fast-track group (n=25, mean age 64±9, 56% females);	TKA	1, 2, 6 and 12 weeks, 1, 2 and 5 years	↓VAS pain scores, LOS, and ↑TUG times, mobility on functional tests, operative time, intraoperative blood loss in fast-track group *vs.* non-fast-track group

			-Non-fast-track group (n=24, mean age 61±7, 62.4% females)			
Gromov et al. 2019 [79]	RCT	OA	29 patients (mean age 64, 62% females):<br><br>-Fast-track group + standard three-layer closure with staples + tissue adhesive group;<br><br>-Fast-track + standard three-layer closure with staples group	Bilateral TKA	72 h, 3 weeks, and 3 months	↓Dressing changes in knees with tissue adhesive. ↑no dressing changes before discharge (59% *vs.* 24%) in knee treated with tissue adhesive *vs.* knee treated without tissue adhesive
Gromov et al. 2020 [80]	Prospective	NR	Fast-track group: 3,927 patients (mean age 66.2, 53.9% females)	UKA	30 and 90 days	LOS=1.3 days. 30- and 90-day readmission rate=4.2% and 6.9%. 90-day mortality=0.08%
Higgins et al. 2020 [88]	Retrospective	OA	1256 patients:<br><br>-Fast-track + PES (n=473, mean age 67.8, 54.4% females);<br><br>-Fast-track group (n=783, mean age 69.4, 59.6% females)	TKA	30 and 60 days	↓LOS (5.2 days versus 6.6 days), ↓rate of reoperation within 60 days (2.2% vs 5.0%) and ↑PROMs in PES patients
Hoorntje et al. 2017 [91]	Retrospective	OA	40 patients:<br><br>-Fast-track (n=20, mean age 63.8±7.5, 65% females);	UKA	3 months	↓HADS (3 *vs.* 8) and ↑NRS in (8 *vs.* 5) in non-fast-track patients

			-Non-fast-track (n=20, mean age 62.2±5.5, 50% females)			
Husted et al. 2011a [93]	Prospective	OA	Fast-track patients (n=29, median age 67, 55.1% females)	Revision TKA	3 months	LOS=(1-4) days; perioperative complications: one anesthesia-related complication, n=7 cortical cracks/fractures, n=1 fractures lines at both stem ends; blood transfusion: n=8, patient satisfaction (verbal analog scale 0-10): 10 (8-10), readmission n=3
Jensen et al. 2020 [98]	Prospective	OA	Fast-track group: 100 patients (mean age 67, 57% females)	UKA	NR	Median LOS: 1 day (range 0-3); discharge: 22% on DOS; 78% on day 1; 98% on day 2; lack of mobilization and pain separately delayed discharge in respectively 78% and 24% of patients on DOS. Main reasons for lack of mobilization: motor blockade (37%) and logistical factors (26%)
Jensen et al. 2021 [99]	Retrospective	OA	Fast-track:	UKA and TKA	90 days	↓Median LOS in UKA than UKA (1 *vs.* 2 days), ↑DOS discharge of UKA (21.1% *vs.* 0.5%),

			-UKA (n=2,786 mean age 66.0, 53.7% female);<br><br>-TKA (n=7,708, mean age 66.5, 54.5% female)			↓prosthetic joint infection and reoperations in UKA, ↑aseptic revisions in UKA
Jiang et al. 2019 [100]	Prospective	OA	256 patients:<br><br>-Fast-track group (n=106, mean age 74.2±6.3, 54.7% females);<br><br>-Non-fast-track group (n=141, mean age 75.4±5.9, 58.8% females)	TKA	1, 3 and 5 days, 1 month, 2 years	VAS: ↓fast-track group at day 1 and day 5; clinical outcomes: ↓fast-track intraoperative blood loss, total blood loss, transfusion rate
Klement et al. 2019 [109]	Retrospective	OA	264 patients (mean age 66.2±9.4, 56.4% females):<br><br>-Fast-tack + FNB + LB-PAI group (n=146);<br><br>-Fast-track + ACC + iPACK group (n=118)	TKA	15 months	ACC + iPACK group: ↓median LOS (2.0 *vs.* 3.0 days); ↓median IV ME 24 h postoperatively; ↑discharge to home; ↓opioid consumption; ↓opioid related adverse events
Lamplot et al. 2014 [114]	RCT	OA	36 patients:<br><br>-Fast-track + multimodal analgesics group	TKA	3 weeks	↓VAS pain score at rest and during physical therapy in multimodal group; ↓total narcotic consumption, daily narcotic consumption,

			(n=19, mean age 66.9, 57.8% females);<br><br>-Fast-track + PCA group (n=17, mean age 62.1, 41.1% females)			narcotic-related adverse events, and time to physical therapy milestones in the multimodal group; ↑satisfaction score in multimodal group
Larsen et al. 2012 [115]	Prospective	OA	Fast-track group: 211 patients (mean age 67, 51% females)	TKA or UKA	4 and 12 months	Average LOS: 3.9 days; ↑HRQOL in comparison to the background population norm at 4 months; ↓SF36 at 4 months and 12 months *vs.* background population
Li et al. 2021b [122]	Prospective	OA	86 patients:<br><br>-Fast-track nursing group (n=43, mean age 67.3±5.2, 34.8% females);<br><br>-Fast-track nursing + limb rehabilitation training group (n=43, mean age 68.1±5.2, 41.8% females)	TKA	30 days	↑Knee joint function, limb motor ability and neurological function, increase patients' cognition of disease and reduce the incidence of complications in fast-track nursing + limb rehabilitation training group compared with fast-track nursing group
Lindberg-Larsen et al. 2017 [123]	RCT	OA	73 patients:<br><br>-Fast-track + MP group (n=33, 65 age, 61% females);<br><br>-Fast-track + isotonic saline group (n=30,	TKA	Until discharge	↓Circulating markers of endothelial activation and damage and systemic inflammatory response (C-reactive protein) in fast-track + MP group

			67.7 age, 50% females)			
Lindberg-Larsen et al. 2018a [124]	RCT	OA	69 patients:<br><br>-Fast-track + MP group (n=33, 65 age, 61% females);<br><br>-Fast-track + isotonic saline group (n=30, 67.7 age, 50% females)	TKA	Until discharge	No differences in orthostatic hypotension and intolerance between groups. ↓C-reactive protein in fast-track + MP group
Lindberg-Larsen et al. 2018b [125]	RCT	OA	122 patients:<br><br>-Fast-track + MP group (n=62, 65 age, 52% females);<br><br>-Fast-track + isotonic saline group (n=60, 67.4 age, 55% females)	TKA	48 h	↑Plasma glucose and insulin resistance and impaired insulin secretion in response to hyperglycemia in fast-track + MP group
Lindberg-Larsen et al. 2019 [126]	Retrospective	OA	464 patients:<br><br>-Fast-track + simultaneous bilateral TKA group (performed under same anesthesia, n=232, 64.6 age, 53.4% females);<br><br>-Fast-track + bilateral TKA group (performed with 1–6	Bilateral TKA	1 months	LOS=4 days, in-hospital complication rate=15.5% in simultaneous group *vs.* 7.3% in bilateral group; 0.9% venous thromboembolic events in each group, re-operated=3.4% after simultaneous *vs.* 0.4% after bilateral TKA; 30-day readmission rate=8.6% in

			months, n=232, 65 age, 53% females)			simultaneous *vs.* 5.6% in bilateral group
Maempel et al. 2015 [129]	Prospective	OA	165 patients:<br><br>-Fast-track group (n=84, 69.8 age, 50% females);<br><br>-Non-fast-track group (n=81, 70.1 age, 54.3% females)	TKA	1 years	LOS=3 days in fast-track and 4 in non-fast-track
McDonald et al. 2012 [131]	Prospective	OA	1816 patients:<br><br>-Fast-track group (n=1081, mean age 69, 59.3% females);<br><br>-Non-fast-track group (n=735, mean age 70, 58.2% females)	TKA	1 years	↓Median LOS in fast-track from 6 to 4 days; ↓post-operative urinary catheterization (35% *vs.* 6.9%) and blood transfusion rates (3.7% *vs.* 0.6%) in fast-track group. Median pain scores on mobilization three throughout hospital stay with 95% of patients ambulating within 24 h in fast-tack group
Munk et al. 2012 [134]	Prospective	OA	Fast-track group: 35 patients (mean age 66, 48.5% females)	UKA	4 weeks	Preoperative level of leg-extension power and functional performance after 1 month
Nicolaiciuc et al. 2019 [136]	Retrospective	Primary or secondary gonarthrosis	108 patients:<br><br>-Fast-track + tournique group (n=53);	TKA	2 and 6 days	↓Pain, oxycodone use, and ↑non-opioids use in fast-track without tournique group *vs.* fast-

			-Fast-track without tournique group (n=55)			track with tournique group
Noel et al. 2020 [137]	RCT	OA	69 patients:<br><br>-Fast-track + oral oxycodone extended-release group (n=34, 67.6±8.2 age, 61.7% females);<br><br>-Fast-track + oral oxycodone immediate-release group (n=35, 65.3±9.0 age, 57.1% females)	TKA	2 days	=Pain and first mobilization
Pamilo et al. 2018 [140]	Retrospective	OA	4256 patients:<br><br>-Fast-track group (n=1061);<br><br>-Non-fast-track group (n=3195)	TKA	2 and 5 years	↓Median LOS from 5 to 3 days and LUIC from 7 to 3 days in fast-track group; ↑in discharge rate to home in fast-track group
Petersen et al. 2020b [144]	Prospective	OA	Fast-track group: 1810 patients (mean age 66.2, 53.9% females)	UKA	90 days	Median LOS 1 day; 7.5% with LOS >2 days. 90-day incidence of VTE=16. 5 pulmonary embolisms, 11 deep-vein thrombosis after median 18 days. 90-day mortality=3

Plessl et al. 2020 [149]	Retrospective	OA	323 patients (mean age 65.7, 63.6% females):<br>-Fast-track group (n=194);<br>-Non-fast-track group (n=129)	TKA	1 years	Mean LOS for the fast-track and non-fast-track groups 0.8 and 2.5 days; fast-track associated with ↑flexion at 2, 6, and 12 weeks and a ↑probability of attaining flexion ≥120° at 6 and 12 weeks; fast-track ↓severe flexion contracture and ↓probability of flexion contracture ≥10° at 2, 6, and 12 weeks
Ruiz et al. 2018 [155]	Prospective	OA	Fast-track group: 50 patients (mean age 66.7, 44% females)	UKA	30 days	94% patients discharged the day of surgery; at 30 days: 80% of patients very satisfied, 10% satisfied and 4% not satisfied
Rytter et al. 2017 [156]	Prospective	OA	72 patients (mean age 65.5, 58.8% females):<br>-Fast-track group (n=37);<br>-Fast-track + single preoperative dose of systemic MP 125 mg group (n=35)	UKA	4 months	↓Pain at rest and during walking, ↓opioids consumption, ↑knee extension in fast-track + MP group
Saku et al. 2019 [157]	Prospective	OA	Fast-track group (n=849, mean age 67.7, 68% females)	TKA	Until discharge	Median LOS: 3 days; risk factors for delayed discharge: age, ↑American Society of Anesthesiologists score,

						general anesthesia, surgery performed toward the end of the week, ↑duration of surgery, ↑stay in the post-anesthesia care unit, and ↓preoperative walking distance
Schotanus et al. 2017 [159]	Prospective	OA	20 patients (mean age 65, 35% females): -Fast-track group (n=10); -Fast-track + OS pathways (n=10)	TKA	6 weeks	Similar early physical activity parameters in fast-track patients and fast-track + OS
Skovgaard et al. 2013 [161]	RCT	OA	24 patients (mean age, 65, 35% females): -Fast-track + saline sprayed in knee joint (n=14); -Fast-track + fibrin sealant sprayed in knee joint (n=10)	Bilateral TKA	7 days	Median LOS: 3 days; no difference between groups in term of output from drains, swelling, strength, pain
van den Belt et al. 2015 [174]	Prospective	OA	Fast-track group: 240 patients (mean age 64.1, 59.6% females)	TKA	At discharge	Median LOS: 5 days; wound, exudate and range of motion at the day of surgery: predictive factors for LOS in fast-track procedures

Van Egmond et al. 2015 [175]	Prospective	OA	Fast-track group: 30 patients (mean age 68, 60% females)	TKA	6 weeks	↓Pain and ↑quality of life and function during the 6 weeks after surgery. Mean h of weekly physiotherapy: 0.6 for the first week and 0.9 during the 6 weeks. Medical consultations in 9 patients during the 6-week
Wang et al. 2018a [21]	RCT	OA	200 patients (mean age 68, 60% females):<br>-Fast-track + single dose of 2.0 g of TXA orally 2 h pre-op (group A);<br>-Fast-track + single dose of TXA followed by 1.0 g orally 3 h post-op (group B);<br>-Fast-track + single dose of TXA followed by 1.0 g 3- and 9-h post-op (group C);<br>-Fast-track + single dose of TXA followed by 1.0 g orally 3-, 9-, and 15-h post-op (group D)	TKA	Until discharge	↓Mean total blood loss in groups C and D *vs.* groups A and B; ↓mean hidden blood loss in groups B, C and D *vs.* in group A; ↓reduction in Hb level in groups C and D *vs.* in groups A and B

Wang et al. 2018b [22]	RCT	OA	147 patients:<br><br>-Fast-track + 3 oral dose TXA (2 g of TXA 2 h before incision, and 1 g of TXA 6 and 12 h after surgery) group (n=74, mean age 65±13.1, 78.37% females);<br><br>-Fast-track + IA TXA (3 g of TXA in 100 mL of saline solution) (n=73, mean age 63.6±11.5 76.71% females)	TKA	3 months	Mean total blood loss: 788.8 mL in the oral TXA group *vs.* 872.4 mL in the IA TXA group; transfusion rates: 4% in oral group and 5% IA group
Wang et al. 2019 [23]	RCT	OA	118 patients:<br><br>-Fast-track + IV TXA at 20-mg/kg 10 min before the surgery and 3 h post-op, and then oral 1 g TXA from post-op day 1 to 14 (group A) (n=59, mean age 63±13.9, 75% females);<br><br>-Fast-track + IV TXA at 20-mg/kg 10 min before surgery and 3 h post-op, and then oral 1 g placebo from day 1 to 14 (group B) (n=59, mean age	TKA	3 months	↓Mean total blood loss in Group A *vs.* in Group B; ↑Hb in Group A *vs.* Group B on day. ↓ecchymosis, morbidity, smaller ecchymosis area in Group A *vs.* Group B; ↓blood coagulation level in Group A *vs.* in Group B on day 1 and day 3; ↓swelling in Group A *vs.* in Group B on day 3 and day 14

			63±13.9, 81.03% females)			
Wied et al. 2015 [182]	Retrospective	OA	359 patients:<br><br>-Fast-track + manipulation under anesthesia (n=21, mean age 64, 33.3% females);<br><br>-Fast-track + non-manipulation under anesthesia (n=338, mean age 69, 63% females)	TKA	3 months	Median LOS: 2 days; prevalence of knee manipulation significantly associated with the achieved knee flexion at discharge
Wynell-Mayow et al. 2018 [184]	Retrospective	OA	Fast-track group: 123 patients (mean age 70, 68% females)	TKA	NR	Median LOS: 5 days; median tourniquet time overall: 74 min and ↓year-on-year from 108 to 60. ↑tourniquet time not associated with ↑LOS, with ↑opioid and other complications
Yu et al. 2018 [189]	RCT	OA	88 patients (mean age 69, 77.27% females):<br><br>-Fast-track + compression therapy group (n=44);<br><br>-Fast-track + no compression therapy group (n=44)	TKA	Until discharge	No differences in swelling, post-op blood loss, pain, range of motion, complications; ↑comfort ratings in non-compression group than compression group in the first 24 h

Zietek et al. 2015 [191]	RCT	OA	62 patients:<br><br>-Fast-track standard rehabilitation program group (n=31, mean age 67.9, 64.5% females);<br><br>-Fast-track intensive rehabilitation program group (n=31, mean age 67.9, 58% females)	TKA	14 days	↓Pain; ↓pain while walking on the second post-op day from 6.1 to a mean of 4.9 in the intensive group and from 6.4 to 5.4 in the standard group; ↓pain at rest from 3.3 to 2.2 for the intensive group and from 4.0 to 3.0 for the standard group; pain at rest at 2 weeks: 2.8 in both groups, pain while walking: 3.0 for the intensive group and 3.4 for standard group
Zietek et al. 2016 [192]	Prospective	OA	Fast-track group (mean age 68.3, 86% females): 100 patients	TKA	6 weeks	Pre-op TNF-α inversely correlated with post-op pain scores during walking and with change of pain at rest during 6 weeks after surgery and directly correlated with a ↑post-op KSS
Hip						
Bernaus et al. 2021 [37]	RCT	OA	63 patients (22.2% females):<br><br>-Fast-track + LIA group (mean age 56.31±9.79);	uncemented THA	4, 8, 24 and 48 h	=VAS, analgesic rescue medication consumption (morphine), first sitting and ambulation time, Hb loss, complications, and LOS

			-Fast-track without LIA group (mean age 61.03±7.23)			
Berthelsen et al. 2017 [38]	RCT	Arthritis	29 patients:<br><br>-Fast-track group (n=15, mean age 71.53, 60% females);<br><br>-Non-fast-track group (n=14, mean age 75.21, 57.1% females)	THR	2 weeks and 3 months	↑Functional status, pain, depression and spouses' caregiver satisfaction in fast-track group *vs.* non-fast-track group
Birznieks et al. 2019 [39]	Prospective	NR	46 patients (58.6% females):<br><br>-Fast-track + prilocaine group (n=22, mean age 57.82±11.41);<br><br>-Fast-track + bupivacaine group (n=24, mean age 55.67±14.09)	THR	NR	↓Pain during movement (3.33 *vs.* 2.00), LOS (~1 day) and ↑patient self-care (90.91% *vs.* 62.5%) in fast-track prilocaine group *vs.* fast-track + bupivacaine group
D'Amato et al. 2019 [49]	Retrospective	OA	211 patients (age range 60-80):<br><br>-Fast-track + tapentadol group (n=106);	THR	1, 2, 3, 4 days	↓Pain (at rest and during movement), supplemental analgesia uses in tapentadol group *vs.* oxycodone/naloxone group; ↑adverse events (PONV, itching, constipation) in

			-Fast-track + oxycodone/naloxone group (n=105)			oxycodone/naloxone *vs.* tapentadol group
Dawson-Bowling et al. 2014 [53]	Retrospective	NR	Fast-track group: 100 patients (mean age 65)	Unilateral THR	6 weeks, 6 and 12 months, 3 years	LOS=1.99 days; 97% patients satisfied; complications n=7
De Ladoucette et al. 2020 [54]	Prospective	Non-traumatic condition	105,855 patients:<br><br>-Fast-track group (n=1,110, mean age 67.5±11.9, 55% females);<br><br>-Non-fast-track group (n=104,745, mean age 69.3±11.6, 57, 54.9% females)	Primary THA	90 days	↓LOS (3.3±2.9 *vs.* 7.5±5.3 days), 90-day readmission (4% *vs.* 11%), and ↑discharge (79% *vs.* 69%) in fast-track group *vs.* non-fast-track group
den Hartog et al. 2017 [59]	Retrospective	OA	Fast-track group: 74 patients (mean age 67.1, 51.3% females)	THA	NR	LOS=1.8 days; pre-op use of pain medication and preoperative neuropathic pain associated with ↑post-op pain
den Hartog et al. 2015 [58]	Retrospective	NR	Fast-track group: 477 patients (mean age 71.0, 66.4% females)	THA	NR	LOS=2.9 (≤2 nights in 266 patients, >2 nights in 211 patients); age, living situation and approach associated with ↑LOS
Ding et al. 2020 [61]	Retrospective	Dysplasia, osteonecrosis, primary OA,	311 patients:<br><br>-Fast-track with LOS ≤3 group (n=196,	THA	90 days	↑Post-op Hb and ↓costs in LOS ≤3 group *vs.* LOS >3 group

		suppurative arthritis, rheumatoid arthritis, fracture, Legg-Calve-Perthes disease, ankylosing spondylitis	mean age 55.7±13.4, 45.9% females);<br><br>-Fast-track with LOS >3 group (n=115, mean age 53.6±16.2, 65.2% females)			
Dwyer et al. 2012 [64]	Prospective	NR	127 patients:<br><br>-Fast-track group (n=64, mean age 70.5, 57.8% females);<br><br>-Non-fast-track group (n=63, mean age 72.5, 65.07% females)	THA	1 and 3 weeks	↓LOS (5.3 *vs.* 8.3 days) in fast-track group *vs.* non-fast-track group
Füssenich et al. 2020 [72]	Prospective	OA	360 patients:<br><br>-Fast-track group (n=185, mean age 68.5±9.9, 58.9% females);<br><br>-Control group (n=175, 66.9±8.1, 58.2% females)	Primary THA	4 weeks, 6 and 12 months	↓LOS (4.4 *vs.* 11.3 days), day hospitalization (4% *vs.* 98%), postoperative (21% *vs.* 92%) and inpatient (21% *vs.* 72%) rehabilitation in fast-track group *vs.* non-fast-track group
Gomez et al. 2019 [75]	Prospective	Hip fractures	54 patients:<br><br>-Fast-track group (n=27, mean age 84.5±8.7, 74% females);	Intramedullary nailing	1 year	↓LOS (2.70±0.91 *vs.* 9.44±4.29 days), costs, operative time (32.90±24.1 *vs.* 55.10±38.7 h) in fast-

			-Non-fast-track group (n=27, mean age 85.0±8.5, 74% females)			track group *vs.* non-fast-track group
Gomez et al. 2020 [76]	Prospective	Hip fractures	54 patients:<br><br>-Fast-track group (n=27, mean age 84.5±8.7, 74% females);<br><br>-Non-fast-track group (n=27, mean age 85.0±8.5, 74% females)	Intramedullary nailing	1 year	↓Parker score, <loss of walking autonomy, without impact on discharge destination or walking aids, in fast-track group *vs.* non-fast-track group
Götz et al. 2021 [77]	Retrospective	NR	Fast-track group: 102 patients (mean age 61.3±10.9, 34.3% females)	THA	4 weeks	↓Pain, ↑ROM, HHS in post-op *vs.* pre-op
Gromov et al. 2015 [78]	Retrospective	Dislocation	253 patients:<br><br>-Fast-track group (n=188, mean age 78, 73.9% females);<br><br>-Non-fast-track group (n=214, mean age 76, 68.2% females)	THA and hemiarthroplasty	NR	↓Surgical delay (2.5 *vs.* 4.1 h), LOS (26 *vs.* 31 h) in fast-track group *vs.* non-fast-track group
Gupta et al. 2014 [81]	Prospective with retrospective control	Fractures	494 patients:<br><br>-Fast-track group (n=259, mean age 81, 74.9% females);	NR	NR	↓Time from emergency department to ward (3.28 *vs.* 10.2 h), LOS (19.6 *vs.* 34 days), and ↑pre-op geriatrician (76.1% *vs.* 4.2%) and falls (73.9%

			-Non-fast-track group (n=235, mean age 82, 71.9% females)			*vs.* 5.1%) assessment in fast-track group *vs.* non-fast-track group
Hansson et al. 2015 [83]	Retrospective	Fractures	441 patients (mean age 84, 72.5% females)	THA and hemiarthroplasty	6 and 12 months	↓Surgery time (62% *vs.* 78%, surgery within 24 h)
Hartog et al. 2015 [84]	Prospective	NR	27 patients (mean age 63, 55.5% females)	THA	3 months	88.8% of patients discharge on day of surgery. ↑PROMs, satisfaction, EQ-5D, and ↓NRS pain (from 6.6 to 1.9); no complications or reoperations
Herndon et al. 2020 [87]	Retrospective	NR	74 patients:<br>-Fast-track + chloroprocaine group (n=37, mean age 61.3±12.5, 67.5% females);<br>-Fast-track + bupivacaine group (n=37, mean age 60.7±9.2, 62.1% females)	THA	NR	↓LOS (0.9 *vs.* 1.2 days), operative time (68.2 *vs.* 83.6 min), EBL (184.7 *vs.* 218.9 mL), PACU LOS (139.4 *vs.* 194.9 min), intra-op hypotension (59.5% *vs.* 83.8%), and ↑discharge (100% *vs.* 89.2%) in chloroprocaine group *vs.* bupivacaine group
Huang et al. 2021 [92]	Retrospective	Fracture	Fast-track group: 1,138 patients (mean age 74.9±8.8, 76.7% females)	THA bipolar HA	Mean 21.2 months, and 3 months, 1, 2 and 3	LOS=2.2 days; 92.4% discharged 48 h after surgery; blood transfusion rate=9.7%; complications=1.4%; readmission rate=1.05%

					years for HHS	
Imbelloni et al. 2014 [96]	Prospective	Fracture	168 patients:<br><br>-Fast-track group (n=85, mean age 75.38±10.53, 70.5% females);<br><br>-Non-fast-track group (n=83, mean age 78.96±10.43, 63.8% females)	Corrective hip fracture	1 day	↓LOS (11.94±6.54 *vs.* 24.77±14.72), surgery suspension number (0.12±0.36 *vs.* 1.25±1.12), fasting time (2.48±0.26 *vs.* 13.38±2.02) in fast-track group *vs.* non-fast-track group
Kang et al. 2019 [105]	Retrospective	Fracture	100 patients:<br><br>-Fast-track group (n=50, mean age 77.81±8.14, 70% females);<br><br>-Non-fast-track group (n=50, mean age 78.32±8.24, 68% females)	PFNA intramedullary fixation	3 and 6 months	↓LOS (from 8.21±0.83 to 5.82±0.64 days), opioid use, opioid-related adverse events, 30-day readmission rates in fast-track group *vs.* non-fast-track group
Klapwijk et al. 2017 [108]	Prospective	NR	Fast-track group: 94 patients (mean age 65, 59.5% females)	THA	6 weeks	LOS=1 night. ↓NRS pain, ICOAP score, pain medication use, HOOS-PS (from 34 to 16), and ↑patients' resistance regarding LMWE injection, walking quality, OHS score (from 29 to 43), EQ-5D (from 0.69 to 0.78)

Kolodziej et al. 2020 [110]	Retrospective	NR	Fast-track group: 30 patients (mean age 60.2, 10% females)	Bilateral THA	Mean 28 months	LOS=4.5 days. ABT in 4 patients (13%)
Larsson et al. 2016 [116]	RCT	Fracture	400 patients:<br><br>-Fast-track group (n=195, mean age 83, 67.6% females);<br><br>-Non-fast-track group (n=205, mean age 82, 65.8% females)	NR	4 months	LOS=between groups; ↓time from arrival to start for RX (28 *vs.* 145 min) in fast-track *vs.* non-fast-track group
Leiss et al. 2021 [117]	Retrospective	NR	Fast-track group: 109 patients (mean age 62.1±10.5, 35.7% females)	THA	4 weeks and 12 months	↑HHS (39.0 *vs.* 21.5), EQ-5D-5L, EQ-VAS, PROMS, satisfaction, and ↓WOMAC, subscale pain (1.5±2.5 *vs.* 11.4±3.6), subscale stiffness (1.08±1.36 *vs.* 4.69±1.74), subscale physical function (5.2±8.2 *vs.* 35.8±12.3) in post-op *vs.* pre-op
Li et al. 2020b [120]	Retrospective	Dysplasia	168 patients:<br><br>-Fast-track group (n=86, mean age 4.0±1.3, 87.2% females);<br><br>-Non-fast-track group (n=82, mean age 4.2±1.3, 84.1% females)	Osteotomy	3 days	↓LOS (6.0±0.8 *vs.* 10.0±3.1 days), VAS score (2.9±0.8 *vs.* 4.0±0.8) in fast-track group *vs.* non-fast-track group

Mikkelsen et al. 2014 [133]	RCT	OA	62 patients:<br><br>-Fast-track + home based exercise 5 days/week and progressive resistance training 2 day/week group (n=32, mean age 64.8, 44% females);<br><br>-Fast-track + home based exercise 7 days/week group (n=30, mean age 65.1, 40% females)	THA	10 weeks	↑Maximal walking speed and stair climb performance in fast-track + home based exercise 5 days/week and progressive resistance training 2 days/week group *vs.* the other group
Okamoto et al. 2016 [138]	RCT	NR	126 patients:<br><br>-Fast-track group (n=58, mean age 62.3±11.6, 38% females);<br><br>-Non-fast-track group (n=68, mean age 62.3±15.2, 41% females)	THA	NR	=LOS; ↑discharge time (63 *vs.* 70 h) in fast-track group *vs.* non-fast-track group
Otte et al. 2011 [139]	Prospective	NR	Fast-track group: 50 patients (mean age 56, 36% females)	Simultaneous bilateral THA	90 days	LOS=4 days; mortality=4%, further operative procedure=8%, complication rate=22%
Pollmann et al. 2016 [150]	Prospective	Fracture	2,230 patients: (mean age 64.1±9.3, 63.6% females):	THA	30 days	↓Admission and surgery time, 30-day reoperation rate, composite 30-day

			-Fast-track group (n=1,140);<br><br>-Non-fast-track group (n=1,090)			outcome (reoperation, surgical site infection and/or death) in fast-track group, but not after adjusting for age, gender, cognitive impairment and ASA score
Porsius et al. 2018 [151]	Prospective	OA	Fast-track group: 94 patients (mean age 65, 56% females)	THA	45 days	Fast (n=17), average (n=53), and slow (n=24) recovery subgroup. Subgroups differed on the estimated weekly growth rate during the first 2 weeks (fast: 9.5; average: 5.3; slow: 2.7), with <differences between groups in the last 4 weeks (fast: 0.90; average: 2.0; slow: 1.7)
Specht et al. 2011 [165]	RCT	OA	60 patients:<br><br>-Fast-track with LIA group (n=30, mean age 68, 60% females);<br><br>-Fast-track with LIA + LINFA group (n=30, mean age 64, 33% females)	THA	7 days	=LOS, pain, tiredness, opioid consumption
Talboy et al. 2016 [170]	Retrospective	Fracture	100 patients (mean age 83, 73% females):	Hemiarthroplasty	At discharge	↓LOS (7 *vs.* 8.5 days), opiate consumption and PCA in fast-track group *vs.* non-fast-track group

			-Fast-track group (n=50);<br><br>-Non-fast-track group (n=50)			
Tan et al. 2018 [171]	Prospective	OA	230 patients:<br><br>-Fast-track group (n=115, mean age 64.6, 67.8% females);<br><br>-Non-fast-track group (n=115, mean age 63.9, 65.2% females)	THA	6 weeks	↓LOS, and ↑% patients with zero oral morphine in fast-track group
Temporiti et al. 2020 [172]	Prospective	OA	71 patients:<br><br>-Fast-track + mobilization and walking the day of surgery group (n=36, mean age 60.9, 57.14% females);<br><br>-Fast-track + mobilization and walking the day after surgery group (n=35, mean age 65.5, 61.7% females)	THA	7 days	↑FIM total and motor scores and FIM self in fast-track + mobilization and walking the day of surgery
Vesterby et al. 2017 [179]	RCT	OA	72 patients (mean age 63.5, 53% females):<br><br>-Fast-track group (n=36);	THA	1 year	↓LOS (from 2.1 to 1.1 days), number of post-op hospital contacts (at 12 months) in telemedicine group

			-Fast-track + telemedicine group (n=36)			
Xie et al. 2019 [185]	Retrospective	Fracture	609 patients (≥65 age, 53% females):<br><br>-Fast-track + prophylactic TXA (n=289);<br><br>-Fast-track no TXA (n=320)	Hemiarthroplasty	3 months	↓LOS, red blood cell transfusion risk (62%), %patients receiving at least 1 U of erythrocytes, and ↑Hb, % patients that ambulate within 24 h after surgery in TXA group *vs.* no TXA group
Zhang et al. 2020 [190]	Prospective	Avascular necrosis	70 patients:<br><br>-Fast-track + clinical nursing pathway group (n=35, mean age 58.77±13.97, 60% females);<br><br>-Clinical nursing pathway group (n=35, mean age 59.66±11.24, 57% females)	THA	3 weeks and 3 months	↑HHS, SF-36 score, satisfaction, ↓complications, hospitalization time in fast-track + clinical nursing pathway group *vs.* clinical nursing pathway
Knee and Hip						
Adams et al. 2021 [26]	Retrospective	Failure of conservative joint pain management	Fast-track group 1200 patients (mean age 62.1, 49.5 females)	SDD-TJA	30 days	↑FTL with general anesthesia, ↓complications and readmission rate
Alvis et al. 2021 [28]	Retrospective	NR	282 patients:	TKA and THA	30 days	↓LOS (2 *vs.* 3 days), opioid use, costs in fast-

			-Fast-track group (n=186, mean age 63.5, 8.6% females);<br><br>-Non-fast-track group (n=96, mean age 65.0, 12.5% females)			track group *vs.* non-fast-track group
Andreasen et al. 2017 [29]	Prospective	NR	Fast-track group: 465 patients	THA and TKA	1 year	Median LOS: 2 days
Awada et al. 2019 [32]	Prospective	NR	Fast-track group: 104 patients (mean age 65.5, 61.5% females)	THA and TKA	0-3 days, 2-3 weeks	↓Incidence of post-op cognitive dysfunction (3.9% patients, n=4)
Berg et al. 2018 [34]	Retrospective	OA	14,148 patients:<br><br>-Fast-track group (n=7345, mean age 69.2±9.6, 56.5% females);<br><br>-Non-fast-track group (n=6803, mean age 69.5±9.8, 57.6% females)	THR and TKR	30 and 90 days	↓LOS (3 *vs.* 5) in fast-track group *vs.* control group. =readmissions and complications
Berg et al. 2020 [35]	Retrospective	OA	55,245 patients:<br><br>-Fast-track group (n=32,558, mean age 68.5±9.5, 56.1% females);<br><br>-Non-fast-track (n=22,687, mean age	THR and TKR	1 year	↑Satisfaction VAS, EQ-5D, EQ VAS, KOOS, and ↓LOS (2-4 *vs.* 4-7 days), VAS pain score in fast-track group *vs.* non-fast-track group

			69±9.5, 57.8% females)			
Berg et al. 2021 [36]	Retrospective	OA	61,571 patients:<br><br>-Fast-track group (n=35,986, mean age 68.2, 68.2% females);<br><br>-Non-fast-track group (n=25,585, mean age 68.6, 57.7% females)	THR and TKR	2 years	↑Risk of revision in THR but not in TKR in fast-track group; ↓TKR or similar THR mortality in fast-track compared to non-fast track group
Bjerregaard et al. 2015 [40]	Prospective	NR	1054 patients (≥18 age):<br><br>-Fast-track catheterized group (n=424, 62,5% females);<br><br>-Fast-track non-catheterized group (n=630, 58.4% females)	THA and TKA	NR	POUR incidence=40%; urinary bladder volume=0.6L; IPSS=6 in non-catheterized males and 8 in catheterized males, 6 in females in both groups. Spinal anesthesia increased the risk of POUR
Bjerregaard et al. 2016 [41]	RCT	NR	721 patients:<br><br>-Fast-track with catheterization threshold 800-ml group (n=367);<br><br>-Fast-track with catheterization threshold 500-ml group (n=354)	THA and TKA	30 days	LOS=2 days; ↓urinary catheterization, without increasing urological complications (13.4% *vs.* 32.2%) in fast-track with catheterization 800-ml group *vs.* fast-track with catheterization 500-ml group

Castle et al. 2021 [43]	Retrospective	NR	386 patients:<br><br>-Fast-track with mobilization <12 h (mean age 69.4±9.1);<br><br>-Fast-track with mobilization ≥12 h (mean age 69±8.6)	THA and TKA	NR	↓LOS (0.5 days) in mobilization <12 h group *vs.* mobilization ≥12 h group (for THA). No reduction in LOS for TKA
Christelis et al. 2015 [45]	Retrospective	OA	709 patients:<br><br>-Fast-track group (n=297, mean age 67±10, 61.9% females);<br><br>-Non-fast-track group (n=412, mean age 68±11, 60.1% females)	TKA and THA	6 weeks	↓LOS (4.9 *vs.* 5.3 days), pain score, urinary catheter removal time and ↑patient satisfaction, patients ready for discharge on day 3 (59% *vs.* 41%) in fast-track group *vs.* non-fast-track group
Davies et al. 2018 [52]	Retrospective	NR	673 patients:<br><br>-Fast-track + TXA group (n=446, mean age 67.3, 54.2% females);<br><br>-Non-fast-track (n=227, mean age 68.7, 66.1% females)	THR and TKR	90 days	↓LOS (5 *vs.* 6 days), costs, blood transfusion (n=28, 6.3% *vs.* n=40, 17.6%), and ↑Hb postoperative in fast-track + TXA group *vs.* non-fast-track group
Drosos et al. 2020 [63]	Retrospective	NR	443 patients:<br><br>-Pre-fast-track + P group;	TKR and THR	30 days	TKR: ↑blood loss, transfusion in non-fast-track and P groups *vs.* P + BMP and P + BMP + PMP groups. ↓LOS (by

			-Pre-fast-track + P + BMP group;<br><br>-Fast-track + P + BMP + PMP group;<br><br>-Non-fast-track group			1.5, 1.56 and 2.1 days) in P, P + BMP and P + BMP + PMP groups *vs.* non-fast-track group.<br><br>THR: ↓LOS (by 1.0, 1.5 and 2.2 days), transfusion in P, P + BMP and P + BMP + PMP groups *vs.* non-fast-track group
Fenelon et al. 2018 [66]	Retrospective	NR	1467 patients:<br><br>-Fast-track group (n=727, mean age 70.7±9.8, 67.3% females);<br><br>-Non-fast-track group (n=740, mean age 70±9.9, 59.9% females)	Primary and revision THA and TKA	NR	↓Intra-op and peri-op blood transfusion, blood loss, costs in fast-track group *vs.* non-fast-track group
Frassanito et al. 2020 [71]	Prospective	OA	Fast-track group: 207 patients	THR and TKR	1, 3 and 6 months	↓LOS (<4 days), and ↑satisfaction (94.4%), KOOS, knee flexion-extension, HOOS, hip abduction (in post-op *vs.* pre-op), ROM
Galbraith et al. 2017 [73]	Retrospective	OA	310 patients:<br><br>-Fast-track group (n=165, 42.4% females);	THA and TKA	30 and 90 days, 1 year	↓LOS (5.1 *vs.* 8.79 days), and ↑discharge in fast-track group *vs.* non-fast-track group

			-Non-fast-track group (n=145, 48.3% females)			
Glassou et al. 2014 [74]	Retrospective	OA	66,733 patients:<br><br>-Fast-track group (n=17,284 procedures, mean age 69±10, 58% females);<br><br>-Non-fast-track group (n=61,814 procedures, mean age 69±10, 59% females)	THA and TKA	90 days	↓LOS, readmission for thromboembolic event, and ↑readmission for infection in fast-track group *vs.* non-fast-track group
Halawi et al. 2019 [82]	Retrospective	OA	378 patients:<br><br>-Fast track without post-op catheterization group (n=213, mean age 60.2±11.2, 46% females);<br><br>-Fast track with post-op catheterization group (n=145, mean age 63.1±12.7, 51% females)	THA and TKA	NR	THA patients: POUR associated with age >60, intra-op fluid volume >1350 mL, and intraoperative placement of an indwelling bladder catheter.<br><br>TKA patients: POUR significantly associated with intra-op indwelling bladder catheter placement
Holm et al. 2014 [89]	Prospective	OA	Fast-track group: 150 patients (THA: n=75, mean age 67±9.1, 58.6% females; TKA: n=75, 65±9.6, 68% females)	TKA and THA	NR	Median discharge readiness and actual LOS until discharge: 2 days. Age as independent predictor of discharge readiness

Husted et al. 2016 [95]	Prospective	OA	Fast-track group: 13,730 procedures	THA and TKA	90 days	LOS=2 days; 30-day readmission rate=6.1% for THA and 5.9% for TKA; 90-day readmission rate=8.6% for THA and 8.3% for TKA
Husted et al. 2012 [94]	Retrospective	OA	NR	THA and TKA	NR	↓LOS from 10-11 days to 4 days
Husted et al. 2011b [12]	Prospective	NR	Fast-track group: 207 patients (mean age 66, 56.5% females)	THA and TKA	6 months	LOS=2.4 days (for TKA group) and 2.2 days (for THA group)
Jørgensen et al. 2021 [103]	Prospective	NR	Fast-track group: 9,987 patients (mean age 70±14, 62.2% females)	TKA and THA	19 months	LOS=1.9 days (80% patients with surgery from Monday to Wednesday, 17% with LOS >2 days *vs.* 19% operated on Thursday and Friday). In high-risk patients, ↑LOS >2 days with surgery on Thursdays or Fridays (43%) *vs.* Monday to Wednesday (37%)
Jørgensen et al. 2017 [102]	Prospective	NR	3,927 patients:<br><br>-Fast-track + MP group (n=1,442, mean age 69, 40.36% females);	TKA and THA	30 and 90 days	↓LOS in fast-track + MP group *vs.* fast-track alone group

			-Fast-track group (n=2,485, mean age 68, 38.2% females)			
Jørgensen et al. 2013b [101]	Prospective	NR	Fast-track group: 3,112 patients (mean age 67, 57% females)	Primary elective THA and TKA	30 and 90 days	LOS=3.0 days; readmission rate=6.6% at 30 days, 9.3% at 90 days; Mortality=0.22% at 30 days, 0.42% at 90 days
Jørgensen et al. 2013a [24]	Prospective	NR	Fast-track group: 5,145 patients (age range 18-97, 56.9% females)	THA and TKA	90 days	LOS=3 days in fallers *vs.* 2 days in patients without falls; injuries: "none" or minor in 39.8%, moderate in 9.6%, major in 50.6%. 54.8% falls within 1 month of discharge. Falls due to physical activity (12.0%) and extrinsic factors (14.5%) occurred later than did surgery-related falls (73.5%), contributing to 40% of all falls 30 days after discharge
Jenny et al. 2020 [97]	Prospective	NR	Fast-track group: 1,949 patients (mean age 70±11, 58.2% females)	THA and TKA	3 months	LOS=3.3±2.9 days for THA and 4.4±3.3 days for TKA; ↑bleeding *vs.* VTE complications (1.7% *vs.* 0.7%) after THA

Krenk et al. 2014 [113]	Prospective	POCD	Fast-track group: 225 patients (mean age 68)	THA and TKA	1-2 weeks and 3 months	LOS=2 days; MMSE score=28; POCD=9.1% (at 1-2 weeks) and 8.0% (at 3 months)
Krenk et al. 2012 [112]	Prospective	OA	Fast-track group: 225 patients (median age 70, 50.6% females)	THA and TKA	12 days	LOS=2.6 days; no patients developed delirium
Kort et al. 2018 [111]	Retrospective	OA	Fast-track group: 638 patients (65.5% females)	THA, TKA, UKA	1 year	POUR=12.9%
Kerr et al. 2017 [106]	Retrospective	OA	Fast-track group: 109 patients (mean age 72, 60.55% females)	THA and TKA	4 weeks	56% of patients discharge at day 5; delay in discharge: oozing wounds (25 patients), medical problems (20 patients), failure to reach physiotherapy goals (14 patients)
Lovecchio et al. 2016 [127]	Retrospective	OA	24,292 patients (age range 60-69):<br><br>-Fast-track inpatients group (n=1,476, 55% females);<br><br>- Fast-track outpatient group (n=492, 56% females)	THA and TKA	30 days	↓Complication rates (1.1% *vs.* 6.3%), transfusion rate (0.1% *vs.* 4.1%) in inpatients group *vs.* outpatients' group
Machin et al. 2013 [128]	Retrospective	OA	226 patients:	THA and TKA	Mean 26.8	↑Patient satisfaction and health scores, recovery in fast-track group *vs.* non-fast-track group

			-Fast-track group (n=101, mean age 62.7);<br><br>-Non-fast-track group (n=125, mean age 66.6)			
Memtsoudis et al. 2020 [132]	Retrospective	OA	1,540,462 patients:<br><br>-High fast-track group (>6 fast-track components, n=324,437, mean age 66, 61.55% females);<br><br>-Medium fast-track group (5-6 fast-track components, n=965,953, mean age 66, 60.41% females);<br><br>-Low fast-track group (<5 fast-track components, (n=250,072, mean age 67, 69.08% females)	TKA and THA	NR	↑Levels of fast-track associated with ↓in 'any complication, mortality, blood transfusions, and length of stay': 'Medium' *vs.* 'Low' and 'High' *vs.* 'Low'; individuals fast-track components with the strongest effect: early physical therapy, avoidance of urinary catheter, TXA administration
Petersen et al. 2017 [141]	Prospective	OA	Fast-track group: 6331 patients (≥70 years)	UKA	90 days	43 cases of post-op delirium that contributed to a LOS >4 days. These patients were older than the patients without delirium by 4.0 years (81 *vs.* 77 years)

Petersen et al. 2019 [142]	Prospective	OA	Fast-track group: 32247 patients (mean age 69, 58% females)	TKA and THA	90 days	↓LOS from a median of 3 days in 2010 to 1 days in 2017. % of LOS >4 days ↓from 9.6% to 4.4%
Petersen et al. 2020a [143]	Prospective	OA	Fast-track group: 1427 patients (mean age 87, 71% females)	TKA and THA	90 days	↓LOS from median 4 days in 2010 to 2 days in 2017. LOS >4 days ↓from 32% to 18%
Petersen et al. 2021 [145]	Prospective	Revision hip and KA	Fast-track group: 2814 patients (mean age 67.7, 63.2% females)	TKA and THA	90 days	Median LOS 3 days; 21% with LOS >5 days. 90-day incidence of VTE: 0.42%, with 0.28% DVT and 0.14% pulmonary embolisms, after median 14 days with the latest on day 31
Pitter et al. 2016 [147]	Prospective	OA	Fast-track group: 522 patients (mean age 87, 74.5% females)	TKA and THA	90 days	LOS >4 days in 27.3%; readmission rates of 14.2% and 17.9% within 30 and 90 days; 75.5% of medical readmissions within 90 days mainly due to falls and suspected but disproved VTE events. 2.0% of 90-day mortality
Plenge et al. 2020 [148]	Prospective	OA	Fast-track group: 186 patients (mean age 62, 68.3% females)	TKA and THA	30 days	Median LOS 4 days; 30-day readmission rate 3.8%; 'days alive and at home up to 30 days after surgery' 26 days; on the first post-op day, out-of-bed mobilization was

						achieved by 38.1% patients, multimodal analgesic regimens administered to 16.0% of patients
Robinson et al. 2014 [153]	Prospective	OA	Fast-track group: 96 patients (mean age 72, 62.5% females)	TKA and THA	Until discharge	Median LOS: 3 days; TXA given to 90% of patients; only 58% of patients received NSAIDs; ↑pain scores in TKA *vs.* in THA
Romano et al. 2021 [154]	Retrospective	OA	181 patients (mean age 71.5, 47.5% females):<br><br>-Fast-track group (n=122);<br><br>-Non-fast-track group (n=59)	TKA and THA	36 months	Fast-track group vs non-fast-track: ↓median LOS, ↑predischarge mean Hb, ↓risk of transfusion, ↓risk of reinfusion, ↑odds ratio of low pain on the first day, ↑risk difference of ambulating the first day, ↓odds ratio of rehabilitation hospital admission, ↑odds ratio of discharge, ↓tourniquet, surgical times, use of catheters and drains. ↓complications at 6, 12, 24, and 36 months in fast-track group
Savaridas et al. 2013 [158]	Prospective	OA	4500 patients (mean age 68.5, 51.2% females):	TKA and THA	24 months	↑Survival probability up to 3.7 years post-surgery in fast-track group

			-Fast-track group (n=1500);<br>-Non-fast-track group (n=3000)			
Stambough et al. 2019 [167]	Retrospective	OA	Fast-track + general anesthesia (n=1527, <55 years: 17%, 55-64 years: 31%, 65-74 years: 34%, >75 years: 19%, 57% females)	TKA and THA	90 days	96.3% of patients discharged on post-op day 1, and 97.2% participate to physical therapy on the day of surgery; 0.4% patients required ICU; 90-day readmission rate 2.4%, reoperation rate 1.3%
Starks et al. 2014 [168]	Prospective	OA	Fast-track group: 2128 patients (mean age 71, 64.6% females)	TKA and THA	Until discharge	Median LOS: 4 days; ↓LOS in patients ≥85: from 9 to 5 days. Readmission rates >45%
Stowers et al. 2016 [169]	Prospective, Retrospective	OA	206 patients:<br>-Fast-track group (n=106, mean age 66.7, 53% females);<br>-Non-fast-track group (n=100, mean age 65.4, 59% females)	TKA and THA	Until discharge	↓Median LOS reduced after fast-track (5 non-fast-track *vs*. 4 fast-track). ↓overall cost in fast-track
Tucker et al. 2016 [173]	Prospective	OA	Fast-track group (n=40 , mean age 66 for THR and 76 for TKR, 57.5% females)	TKA and THA	2 day	Median LOS for THA 1.8 days and for TKA 1.9 days; ↓pain score

Van Horne et al. 2019a [176]	Retrospective	OA	Fast-track group (n=601, mean age 72, 56.7% females)	TKA and THA	30 days	84.0% of patients discharged the same day, 13.8% in 1 day, 2.2% in >1 day; rates of minor and severe adverse events within 30 days: 0.5% and 1.1%, respectively. 1.9% of patients with unplanned readmissions within 30 days; ↑patient-reported satisfaction; 84.2% patients do not require >1 seven-day opioid
Van Horne et al. 2019b [177]	Retrospective	OA	Fast-track group (n=220, mean age 58, 49% females)	TKA and THA	60 days	Within 30 days 2.8% patients with an adverse event; within 60 days, 3.2% patients with an emergency department/urgent care visit. 82.1% patients without a second opioid prescription; ↑patient satisfaction
Winther et al. 2015 [183]	Retrospective	OA	Fast-track group (n=920, mean age 58, 49% females)	Primary and revision TKA and THA	1 years	Mean LOS: 3.1 days for primary patients, 4.2 days for THA revision and 3.9 for TKA revision; mean patient satisfaction: 9.3 out of a max. of 10; revision rates until 1-year follow-up: 2.9% and 3.3% for primary hip and knee patients, and 3.7%

						and 7.1% for revision hip and knee patients
Xu et al. 2019 [186]	Prospective	OA and inflammatory arthritis	6325 patients (mean age 66.60±8.75, 78.59% females):<br><br>-Fast-track + drain (n=4540);<br><br>-Fast-track no drain (n=1785)	TKA and THA	NR	Drain use correlated significantly to a ↑transfusion rate and a longer LOS
Yanik et al. 2018 [188]	Retrospective	OA	252 patients:<br><br>-Fast-track group (n=78, mean age 66.56, 10.3% females);<br><br>-Non-fast-track group (n=174, mean age: 65.89, 9.8% females)	TKA and THA	90 days	↓Average LOS in fast-track group from 3.2 to 1.7 days; ↑of 12.3% in patients discharge in fast-track group

Abbreviations: OA = osteoarthritis; n = number; TKA = total knee arthroplasty; ↑ = increase; vs. = versus; NR = not reported; LOS = hospital length of stay; DVT = deep vein thrombosis; min = minutes; ↓ = decrease; h = hours; NRS = numerical rating scale; RCT = Randomized controlled trial; VAS = visual analog scale; TNF-α = Tumor necrosis factor-alpha; SOD-1 = superoxide dismutase-1; Mb = myoglobin; PTX3 = Pentraxin 3; CCL2 = C-C Motif Chemokine Ligand 2; PGE2 = Prostaglandin E2; ROM = range of motion; MRC = Medical Research Council; TKR = total knee replacement; Hb = hemoglobin; PACU = post-anesthesiological care unit; MME = Morphine Milligram Equivalents; IV = intravenous; TXA = tranexamic acid; TUG = Timed Up and Go test; UKA = unilateral knee arthroplasty; PROMS = Patient-reported outcome scores; PES = pathway management solution; HADS = Hospital Anxiety and Depression Scale; DOS = day of surgery; FNB = femoral nerve block; LB-PAI = liposomal bupivacaine pericapsular injection; ACC = adductor canal catheter; iPACK = posterior capsule single shot block; ME = morphine equivalents; PCA = patient-controlled analgesia; HRQOL = Health Related Quality of Life; SF-36 = 36-item Short Form Health Survey; MP = methylprednisolone; LUIC = length of uninterrupted institutional care; VTE = venous thromboembolism; OS = outpatient surgery; pre-op = preoperative; post-op = postoperative; IA = intra-articular; KSS = Knee Society Score; LIA = local infiltration analgesia; THA = total hip arthroplasty; THR = total hip replacement; PONV = postoperative nausea and vomiting; HHS = Harris hip score; EQ = Euro Quality of Life; EBL = estimated blood loss; HA = hip arthroplasty; PFNA = proximal femoral nail anti-rotation; ICOAP = intermittent and constant osteoarthritis pain score; HOOS-PS = hip injury and osteoarthritis outcome score physical function short form; LMWE = low-molecular-weight eparin; OHS = Oxford hip score; ABT = allogeneic blood transfusions; WOMAC = Western Ontario and McMaster University osteoarthritis index; ASA = American Society of Anaesthesiologists; LINFA = local infusion analgesia; FIM = Functional Independence Measure; SDD = same-day discharge; TJA = total joint arthroplasty; FTL = failure to launch; KOOS = Knee injury and Osteoarthritis Outcome Score; POUR = postoperative urinary retention; IPSS = international prostate symptom score; P = physiotherapy and rehabilitation; BMP = blood management program; PMP = pain management program; intra-op = intraoperative; HOOS = Hip disability and

Osteoarthritis Outcome Score; POCD = postoperative cognitive dysfunction; MMSE = Mini Mental State Examination; KA = knee arthroplasty; NSAIDs = nonsteroidal anti-inflammatory drugs; ICU = intensive care unit.

Table S7. Fast-track components of included literatures studies on orthopedic surgery.

References	Fast-track		
	**Preoperative**	**Intraoperative**	**Postoperative**
Spine			
Adeyemo et al. 2021 [27]	Behavioral health, psychology, nutrition and mineral metabolism, patient education, geriatrics	TXA, controlled epidural analgesia, normotension, transfusional protocols	Early mobilization, multimodal analgesia, thromboprophylaxis, nutrition protocol

	consultation (>65), physical therapy		
Dagal et al. 2019 [51]	Patient education, nutritional support, carbohydrate loading	GDHM, blood loss control (PPV, SVV, or CO), antifibrinolytics	Opioid-sparing multimodal analgesia (acetaminophen, gabapentin, ketamine)
d'Astorg et al. 2020 [50]	Patient education	Opioid-sparing multimodal approach, early catheters and drains removal	Early mobilization, follow-up phone call (day 1), surgical consultation (4-6 weeks)
Debono et al. 2019 [55]	Patient education, infection prophylaxis	Analgesia, no drain, opioid-sparing multimodal approach	Early mobilization, online/phone survey
Debono et al. 2021 [56]	Consultation, online pre-admission and education, anti-infection prophylaxis	Disinfection protocol, no premedication, drain and neck brace, pre-emptive analgesia	Early mobilization, surgical consultation, satisfaction phone survey, online clinical evaluation
Feng et al. 2019 [67]	Patient education, fasting carbohydrate loading (6 h for liquids, 8 h for solid food and short-chain polypeptides drinks, 2 h for clear liquids), pre-emptive analgesia (oral celecoxib 200 mg and pregabalin 150 mg 1 h before)	Antimicrobial prophylaxis (1.5 g cefuroxime 1 h before), TXA (30 min before), normothermia and normovolemia, LIA	Early mobilization, multimodal analgesia (IV parecoxib 40 mg, oral celecoxib 200 mg, pregabalin 75 mg every 12 h, intramuscular tramadol 100 mg), early nutrition
Fletcher et al. 2020 [68]	Consulting	NR	Early mobilization, feeding, catheters and drains removal (day 1)
Fletcher et al. 2021 [69]	NR	NR	NR
He et al. 2020 [85]	TXA IV 10 mg/kg (15 min before)	General anesthesia, TXA IV 6-8 mg/kg/h (up to 15 mg/kg)	Early drainage removal (<30 ml for 24 h)

Heo et al. 2019 [86]	Patient education, emotional support, pre-emptive analgesic (pregabalin 75 mg or gabapentin 300 mg), antibiotics (cephalosporin), IV TXA and antiemetics (PONV prevention)	Local anesthetic, IV TXA and antibiotics, drainage catheter (epidural hematoma prevention), vancomycin	Early mobilization, pain control (IV PCA, oral analgesic w/ pregabalin or gabapentin), thromboprophylaxis, intermittent legs pneumatic compression orthosis, nutrition
Julien-Marsollier et al. 2020 [104]	Patient information	Dexmedetomidine and ketamine	Early mobilization, PCA, opioid-sparing pharmacological and non-pharmacological techniques, feeding, oral medication and urinary catheters and drains removal
Kilic et al. 2020 [107]	Patient education, no smoking, antimicrobial skin cleaning, decreased stress, physiologic function maintenance, clear fluids 2 h and solid food 4 h before, pain management (oral gabapent 300 mg and acetaminophen 1000 mg)	Antibiotic prophylaxis, LMWE, IV 0.15 mg/kg ondansetron and 0.2 mg/kg dexamethasone, TIVA, normothermia, IV 1.5 g TXA and topically 1 g in 100 mL saline, fluid and blood transfusions only with Hb <8 g/dL, 30 cc marcaine hydrochloride 0.5%, no catheters and drains	Early mobilization, opioid-sparing approach (acetaminophen, NSAIDs or tramadol), food and drink intake
Li et al. 2018 [118]	Patient education, no bowel preparation, fasting 6 h and water 2 h before, PONV and antithrombotic prophylaxis	Local anesthesia (0.75% ropivacaine), multimodal analgesia, IV NSAIDs (40 mg parecoxib every 12 h or 100 mg flurbiprofen for 3 days and oral 100 mg celecoxib)	Early mobilization, less infusion volume (1000 ml x 2 days), early oral food intake, catheters and drainage removal
Li et al. 2020a [119]	Patient education, nutritional counselling, clear fluids and carbohydrate drink up to 2 h before, thromboprophylaxis, antimicrobial/antibiotic prophylaxis	TXA, TIVA (propofol, lidocaine, ketamine, ketorolac, antiemetics and anesthetics), LIA, multimodal analgesia, euvolemia, normothermia (36–37°C)	Early mobilization, multimodal analgesia, oral feeding and bladder catheter removal

Li et al. 2021a [121]	Patient education, nutritional counselling, clear fluids and carbohydrate drink up to 2 h before surgery, thromboprophylaxis, antimicrobial/antibiotic prophylaxis	TXA, TIVA (propofol, lidocaine, ketamine, ketorolac, antiemetics and anesthetics), LIA, multimodal analgesia, euvolemia, normothermia (36–37°C)	Early mobilization, multimodal analgesia, oral feeding and bladder catheter removal
Nazarenko et al. 2016 [135]	Patient preparation	Regional anesthesia	Short-acting anesthetics, early rehabilitation
Shaw et al. 2021 [160]	NR	Methadone (29.5 MME, 0.5 MME/kg or 0.1 mg/kg)	Morphine PCA (0.02 mg/kg), oral hydrocodone and acetaminophen (5-10 mg/325mg), ketorolac (0.5 mg/kg), 300 mg gabapentin and diazepam (2 mg)
Soffin et al. 2019b [162]	Patient education, fasting and carbohydrate loading, oral pre-emptive analgesia (1000 mg acetaminophen and 300 mg gabapentin), PONV (1.5 mg scopolamine transdermal)	TIVA (50–100 mg/kg/min propofol and 0.1–0.5 mg/min ketamine), isoflurane or sevoflurane, antimicrobial and antiemetic prophylaxis (IV 4 mg ondansetron, 4-8 mg dexamethasone), normovolemia, normothermia, non-opioid analgesia (15–30 mg ketorolac, 1–2 mg/kg/h lidocaine), no drains/ catheter	Early mobilization, no IV fluid and nutrition (oral diet), opioid-sparing multimodal analgesia
Soffin et al. 2019a [20]	Patient education, oral intake, pre-emptive analgesia (1000 mg acetaminophen, 300 mg gabapentin), PONV (1.5 mg transdermal scopolamine)	Opioid-sparing anesthesia and analgesia, PONV, normothermia (36.0-37.0°C), normovolemia (IV 10–15 ml·kg−1 fluid), antibiotics, no drain or catheter	Early mobilization, early oral intake, opioid-sparing multimodal analgesia (acetaminophen, NSAIDs, tramadol, oxycodone)
Soffin et al. 2019c [163]	Patient education, oral 1000 mg acetaminophen and 300 mg gabapentin	General anesthesia, premedication (midazolam 0.05 mg/kg), propofol (50–150 μg/kg/h), ketamine (0.1–0.5 mg/min), lidocaine (2 mg/kg/h),	Feeding, nonopioid analgesics (acetaminophen, ketorolac,

		isoflurane or sevoflurane, antiemetic therapy (4-8 mg dexamethasone, 4 mg ondansetron, 15-30 mg ketorolac)	gabapentin), tramadol or oxycodone, PONV
Soffin et al. 2020 [164]	Patient education, fasting and carbohydrate loading, pre-emptive analgesia (oral 300 mg gabapentin, 1000 mg acetaminophen within 60 min), PONV (scopolamine patches)	PONV (4–8 mg dexamethasone, ondansetron), IV multimodal anesthesia (propofol, dexmedetomidine, ketamine), analgesia (ketorolac, lidocaine, ketamine), antimicrobial prophylaxis, normothermia (36-38°C) and normovolemia	Early mobilization and nutrition, constipation and ileus prevention, opioid-sparing multimodal analgesia (acetaminophen, ketorolac, gabapentin, tramadol, dextromethorphan)
Staartjes et al. 2019 [166]	Patient education, no smoking and alcohol, nutritional counseling, antimicrobial and antithrombotic prophylaxis	General anesthesia (propofol, sufentanil), LIA (2.5 mg/ml ropivacaine intramuscularly), vasopressors, muscle relaxants use limited, normothermia	Early mobilization, opioid-sparing analgesia (NSAIDs, paracetamol, patient-controlled w/ short-acting opioids), early drains and urinary catheters removal, solids and fluids intake (day 0)
Venkata et al. 2018 [178]	Patient education and information	General anesthesia, antibiotic prophylaxis (1.5 g cefuroxime), no drain or catheter	Early mobilization multimodal analgesia
Wang et al. 2020 [180]	Patient education and counseling, no prolonged fasting, antimicrobial prophylaxis	Anesthetic protocol	Early mobilization, multimodal analgesia, antithrombotic prophylaxis, gastrointestinal management, early feeding and bladder catheter removal
Yang et al. 2020 [187]	Patient education (procedure, LOS, recovery), diet (clear liquid), neurontin (30 min before)	Intrathecal morphine	Early mobilization, multimodal analgesia (patient-controlled, discontinued day 1, and oral oxycodone, valium, neurontin, tylenol, toradol), high fiber diet, neurontin

Thorax			
Holmes et al. 2019 [90]	Patient education	Lidocaine infused bilateral paravertebral catheters (until 2 to 3 days after discharge), PCA (hydromorphone, fentanyl or morphine), ketorolac, acetaminophen, gabapentin and transdermal scopolamine	Early ambulation, catheter removal, general diet, no bowel movement, PCA discontinuation, oral pain medications
Mangat et al. 2020 [130]	Carbohydrate drink (2 h prior), fluids (500-1000 ml), celecoxib (100-200 mg), gabapentin (15-900 mg), acetaminophen (15-1000 mg)	Maintenance anesthetic and fluids, epidural loading and infusion, ketamine, dexmedetomidine, ketorolac	Epidural infusion, morphine or hydromorphone, diet, acetaminophen, gabapentin, ibuprofen, oxycodone
Pirsaharkhiz et al. 2020 [146]	NR	ESP block, general anesthesia, catheter on ipsilateral chest (4-14 days), 20 ml 0.2% ropivacaine and continuous infusion	NR
Wharton et al. 2020 [181]	Patient education, consulting, exercise, gabapentin (300 mg), bowel clean (8.5 g polyethylene glycol)	General anesthesia	Early mobilization, acetaminophen, ibuprofen, oxycodone/hydromorphone, gabapentin, bowel clean, NSAIDs
Elbow			
Cui et al. 2019 [48]	Patient education	Brachial plexus anesthesia, antibiotic prophylaxis, TXA	Early mobilization multimodal analgesia (acetaminophen, NSAIDs, IV patient controlled, low-dose opioids), celecoxib (for 6 weeks), physical therapy and cryotherapy (for 3 days)
PrkiĆ et al. 2020 [152]	Patient education	NR	Exercise, physiotherapy, no cast, functional discharge criteria

Hip and/or knee			
Aasvang et al. 2016 [25]	Opioid use (for at least 4 weeks before), multimodal analgesia (slow-release paracetamol 2 g, celecoxib 400 mg, gabapentin 600 mg, 1-2 h before)	Spinal anesthesia (1.5 ml bupivacaine 0.5%, 1-5 mg/kg/h propofol), LIA (100 ml 0.2% ropivacaine, 10 mg/ml epinephrine, 50 ml 0.2 ropivacaine)	Slow-release paracetamol 2 g, celecoxib 200 mg and gabapentin 300 mg (on the first evening after), sufentanil and morphine
Ascione et al. 2020 [30]	Patient education, information	Spinal anesthesia (2 ml hyperbaric bupivacaine 0.5%, 10 mg/ml propofol), IV TXA (500 mg), local anesthetic (150 ml ropivacaine, 10 mg/ml epinephrine), fluids, compression bandages and cooling, no drains	Early mobilization, oral medication (1 g paracetamol, 200 mg celecoxib, 300 mg gabapentin, opioids), enoxaparin 20 mg subcutaneously
Auyong et al. 2015 [31]	Patient education, care companion, transdermal scopolamine patch	Spinal anesthesia (mepivacaine), 2 L crystalloid fluid, IV TXA (1 g), dexamethasone (4 mg)	Continuous adductor canal nerve block, physical therapy, oral analgesics (acetaminophen, NSAIDs, gabapentin, oxycodone)
Bandholm et al. 2014 [33]	Patient information	NR	Strength training
Cao et al. 2020 [42]	NR	General or local anesthesia, intra-articular drain (prior to closure), tourniquet	Early mobilization, physiotherapy, drain removal
Castorina et al. 2018 [44]	Patient education	IV TXA 5 ml/500 mg, chirocaine 7,5 mg/ml, no drain	Early mobilization, elastomeric pumps infusion (betamethasone, ondansetron, ketorolac, morphine), cryotherapy
Collett et al. 2021 [47]	Preoperative analgesia	Spinal anesthesia, minimal opioids use, bupivacaine HCl 0.25% Ketorolac 30 mg/ml Clonidine HCl	Early mobilization, 30 dose of hydrocodone

		0.1 mg/ml NaCl 0.9% Liposomal bupivacaine 266 mg/20 ml	
Concina et al. 2019 [46]	IV cefazoline 2 g and TXA 15 mg/kg	Tourniquet (300 mmHg, released before closure), suction drain, antalgic therapy (60 mL ropivacaine 7.5%, IV 1 g paracetamol every 8 h, oral oxycodone/naloxone 10/5 mg every 12 h)	Thromboprophylaxis (enoxaparine 4000 IU, 12 h after), IV TXA 15 mg/kg (4 h after), suction drain removal (day 1)
Deiter et al. 2020 [57]	Patient education, oral celecoxib 200 mg, oxycodone 10 mg, acetaminophen 1000 mg	Spinal anesthesia (1.8 mL 0.75% hyperbaric bupivacaine peri-articular infiltration), single shot ACB (20 mL 0.5% ropivacaine), 30 mL 0.5% bupivacaine, toradol 60 mg, 20 mL 0.9% normal saline	Multimodal analgesia protocol (acetaminophen 500 mg every 6 h, toradol 30 or 15 mg every 6 h, oral roxicodone 5 mg every 4 h and tramadol 50 mg every 4 h, IV dilaudid 0.5 mg), physical therapy
Didden et al. 2019 [60]	Patient education and information	LIA (ropivacaïne 2% 150ml morphine-sulphate 5 mg, adrenaline 0.5 mg), spinal or general anesthesia, paracetamol 1000 mg, gabapentin 300 mg (or 100 mg ≥70years/creatine 30–60 ml/min/1.73 m^2), naproxen 500 mg, gastric protector	Early mobilization, gabapentin (2×300 mg or 2×100 mg), paracetamol (4×1000 mg), naproxen (2×500 mg), oxycodone (immediate-release, 6×5 mg and prolonged-release, 2×10 mg), PCA
Drosos et al. 2016 [62]	NR	Spinal anesthesia, pneumatic tourniquet, IV (1 g) or topical (1 g in 30 ml normal saline) TXA, antibiotic prophylaxis, anticoagulation regime, drain	Early mobilization (day 1) and drain removal (24 h after), ABT regime (if Hb < 10.0 g/dl)
Dwyer et al. 2014 [65]	Patient education and information, pre-assessment check, calorie and carbohydrate loading, no premedication	Spinal or epidural anesthesia (propofol), tourniquet use, PONV prevention, short-acting opiates, paracetamol, codeine, anti-	Early mobilization and nutrition, non-opiate and non-steroidal analgesia, oral fluids, thrombo- and infection prophylaxis, vitamin

		inflammatory analgesia, fluids, drain, no urinary catheter	supplements, drain removal (day 1)
Fransen et al. 2018 [70]	Premedication (paracetamol 1000 mg, temazepam 10 mg)	General anesthesia, LIA, no steroids, tourniquet, pain pumps, drain and bladder catheter	Early mobilization, thromboprophylaxis (fraxiparine 2850 international units once a day for 4 weeks), paracetamol 1000 mg (4 times a day), diclofenac 50 mg (3 times a day), oral oxynorm 5 mg, ice packs
Gromov et al. 2019 [79]	IV TXA (1 g)	Tissue adhesive, no drains or tourniquets	Early mobilization, compression bandage removal, standard pain treatment, thromboprophylaxis (10 mg rivaroxaban 6-8 h prior), IV TXA (1 g, 3 h after)
Gromov et al. 2020 [80]	Questionnaires, high-dose corticosteroids, IV TXA	Spinal anesthesia, opioid-sparing multimodal analgesia, no drains	Early mobilization, thromboprophylaxis
Higgins et al. 2020 [88]	PES patients: assess to an online educational platform on general health information, basis of OA and joint pain, each stage of the pathway. Preoperative recording of EQ-5D Index, OKS and CACI	General anesthesia + regional nerve block reserved as a second line for patients in whom spinal was unsuitable, perioperative anticoagulation (TED stockings and enoxaparin 40 mg)	No-PES and PES: early mobilization, pain management, regular long-acting opiates with short acting opiate for pain. PES only: videos exercise, timeline activity, reminders for appointments, provider team online system, questionnaires, streamlined patient pathway
Hoorntje et al. 2017 [91]	Paracetamol (1 g), meloxicam (15 mg), pantoprazole (40 mg) and gabapentin (300 mg) 1 h before surgery	Spinal or general anesthesia, TXA (1 g) at pre-operation and at wound closure, LIA, dexamethasone (8 g)	Early mobilization, cefazolin for 24 h, meloxicam (15 mg once daily) and paracetamol (1000 mg four times daily), oxycodone (10 mg)

Husted et al. 2011a [93]	NR	Spinal anesthesia (3 mL 0.5% (15 mg) plain bupivacaine), propofol (0.5–5 mg/kg/h) if required, TXA (1 g), LIA (300 mg ropivacaine (0.2%), epinephrine (10 mg/mL), fluid administration: 0.9% saline (5 mL/kg/h) and colloid (Voluven 7.5 mL/kg/h), compression bandage	Early mobilization, xarelto (10 mg) compression bandage removal, few h in PACU opioid-spearing analgesia (celecoxib 200 mg), acetaminophen (2 g), gabapentin (300 mg morning and 600 mg evening)
Jensen et al. 2020 [98]	IV injection of 125 mg MP 30 min before surgery together with 2 g of dicloxacillin	Spinal anesthesia (2 mL 0.5% hyperbaric bupivacaine) or general anaesthesia (remifentanil and propofol), LIA (200 mL 0.2% ropivacaine), compression bandage, RX	Early mobilization, paracetamol 1 g x 4 and celecoxib 200 mg x 2 daily for 7 days, no opioids, morphine 5 mg given as rescue medication, RX
Jensen et al. 2021 [99]	Preoperative high-dose corticosteroids, IV TXA	Spinal anesthesia, multimodal opioid-sparing analgesia	Early mobilization, thromboprophylaxis
Jiang et al. 2019 [100]	Quadriceps exercise, knee mobility exercise, ankle pump exercise, lung function exercise, solid diet up to 2 h before surgery	Spinal (dexamethasone 4 mg) or general anesthesia, TXA (1 g), controlled blood pressure, “cocktail therapy” (0.9% sodium chloride, ropivacaine (150 mg), ketorolac (30 mg), epinephrine (0.1 mg)	Early mobilization, multimodal oral analgesia (parecoxib 40 mg a day), early initiation of oral intake
Klement et al. 2019 [109]	Single shot FNB + LB-PAI or ACC + IPACK	Tourniquet, TXA or Amicar	Multimodal analgesia: NSAID, gabapentinoid, analgesic/antipyretic, short acting opioid, long-acting opioid, opioid breakthrough, dexamethasone day 0 and 1
Lamplot et al. 2014 [114]	5 mg cumadin, vancomycin and cefazolin 60 min before surgery	Epidural or spinal anesthesia. Multimodal group: 30 cc 0.5% bupivacaine, 10 mg MSO_4 and 15 mg	Early mobilization, IV morphine or hydromorphone. Multimodal group: oxycodone 10 mg, tramadol 50 mg, ketorolac 15 mg,

		ketorolac. PCA group: no periarticular injection	hydrocodone 5 mg, hydromorphone 1 mg as needed, ondansetron 4 mg, metoclopramide 10 mg. PCA group: hydromorphone 1 mg, ondansetron 4 mg, metoclopramide 10 mg
Larsen et al. 2012 [115]	Screen by a nurse at the first visit	Start mobilization	Early mobilization, analgesia: opioids, NSAID, paracetamol, discharge to home
Li et al. 2021b [122]	Patients' information, preoperative stress relief, psychological counseling, analgesia, water and liquid food 8 h before the operation	NR	Drainage tube removed within 24 h after surgery, local ice compress for 24 h at 24 h after surgery, dietary intervention, infection prophylaxis
Lindberg-Larsen et al. 2017 [123]	Screening for eligibility, enrollment, allocation	Spinal anesthesia, local analgesia, TXA 1 g, fluid therapy (if surgery was prolonged beyond 1 h), no tourniquet	Early mobilization, thromboprophylaxis, no ice packings or mechanical calf compression, analgesia, opioids on request
Lindberg-Larsen et al. 2018a [124]	Screening for eligibility, enrollment, allocation	Spinal anesthesia, local analgesia, TXA 1 g, fluid therapy (if surgery was prolonged beyond 1 h), no tourniquet	Early mobilization, thromboprophylaxis, no ice packings or mechanical calf compression, analgesia, opioids on request
Lindberg-Larsen et al. 2018b [125]	Screening for eligibility, enrollment, allocation	Spinal anesthesia, local analgesia, TXA 1 g, fluid therapy (if surgery was prolonged beyond 1 h), no tourniquet	Early mobilization, thromboprophylaxis, no ice packings or mechanical calf compression, analgesia, opioids on request

Lindberg-Larsen et al. 2019 [126]	NR	Spinal anesthesia, TXA, opioid-sparing analgesia with acetaminophen, NSAIDs	Early mobilization, thromboprophylaxis
Maempel et al. 2015 [129]	Patients' assessment	Spinal anesthesia, analgesia, TXA	Early mobilization, analgesia
McDonald et al. 2012 [131]	Pre-operative education, analgesia	Spinal anesthesia, TXA (2.5 g), multimodal pre-medication, catheter	Early mobilization, catheter remotion, multimodal analgesia, RX and routine blood test on day 2 post surgery
Munk et al. 2012 [134]	Multidisciplinary education, multimodal pain treatment	Spinal or general anesthesia, LIA, TXA (1 g), no drains, elastic compression bandage, thromboprophylaxis	Early mobilization
Nicolaiciuc et al. 2019 [136]	Premedication (40 mg pantozole, 500 mg naproxen, 300 mg gabapentin, 1000 mg paracetamol)	Total IV or spinal anesthesia, LIA (natropin 0.1% 2 x 50 ml with suprarenin, 1 x 50 ml without suprarenin), tourniquet in fast-track + tournique group	Physiotherapy (day 1), oxygesic (10 mg), naproxen (500 mg), paracetamol (1000 mg)
Noel et al. 2020 [137]	Anxiolytic premedication	Spinal anesthesia, multimodal analgesia, IV TXA (1 g), TXA 3 g injection diluted in 70 mL of NaCl 0.9% (after surgery)	Early mobilization, multimodal pain management, PONV
Pamilo et al. 2018 [140]	NR	NR	NR
Petersen et al. 2020b [144]	Patient questionnaires	Spinal anesthesia, multimodal opioid sparing analgesia	Early mobilization, in-hospital thromboprophylaxis if LOS ≤5 days, no pneumatic compression devices or compression stockings

Plessl et al. 2020 [149]	Multimodal pain management	Spinal anesthesia with a single adductor block	Early mobilization
Ruiz et al. 2018 [155]	Pre-operative education	Antibiotics prophylaxis, dexamethasone and TXA 30 min before incision, local analgesia, no drain or epidural catheter, compression bandage	Early mobilization, oral pain medication (NSAID), short acting opioid, thromboprophylaxis
Rytter et al. 2017 [156]	Analgesia, antibiotics prophylaxis, and in fast-track + MP group also a single dose of MP 125 mg IV	Spinal (90% of patients) or general (10%) anesthesia, no drains, analgesia, TXA, compression bandage, cooling device	Early mobilization, thromboprophylaxis, paracetamol, celecoxib, gabapentin, and morphine for pain
Saku et al. 2019 [157]	Pre-operative education	General (9%) and local (91%) anesthesia, analgesia (ropivacaine, adrenalin, ketorolac), TXA	Early mobilization, discharge pain medication, thromboprophylaxis
Schotanus et al. 2017 [159]	Fast-track group: admission night before/day of surgery. Fast-track group + OS: admission day of surgery	Fast-track group: IV prophylactic antibiotics, adrenaline, dexamethasone, TXA. Fast-track group + OS: IV and oral prophylactic antibiotics, dexamethasone, TXA	Fast-track group: first mobilization < 6 h, compression bandages 24 h postoperative. Fast-track group + OS: first mobilization < 4 h, compression bandages 8 h postoperative, first 4 days postoperative elastic bandage
Skovgaard et al. 2013 [161]	Preoperative cefuroxime, TXA, local analgesia	Spinal anesthesia, light sedation, fluid administration, transfusion if Hb < 6.0 mmol/L, drains for 24 h	Early mobilization, multimodal opioid sparing, oral analgesia
van den Belt et al. 2015 [174]	Preoperative information and online “knee portal”	Spinal anesthesia, analgesia, no drain, thromboprophylaxis	Early mobilization, antibiotics, multimodal pain control medication, compression bandage
Van Egmond et al. 2015 [175]	Preoperative education	Spinal anesthesia with local infiltration anesthesia, pain	Early mobilization

		medication, opioid medication only on request, no drains, no urine catheters	
Wang et al. 2018a [21]	Pre-op education, medical risks evaluation, high protein diet and multivitamin supplements, carbohydrate-rich drinks on the night before and of the morning of surgery, TXA (group A)	Stop to solid food for 6 h or clear fluids for 2 h prior to surgery, general anesthesia, multimodal analgesia, thromboprophylaxis	Early mobilization, TXA (group B, C, D)
Wang et al. 2018b [22]	Pre-op education, standard analgesia	General anesthesia, multimodal analgesia, TXA, antibiotic prophylaxis, no tourniquet, IA drainage, pressure dressing, thromboprophylaxis	Post-op laboratory values, TXA
Wang et al. 2019 [23]	Pre-op education, standard analgesia, doppler ultrasound	General anesthesia, no tourniquet, drains, pressure dressing, thromboprophylaxis, TXA	Early mobilization, doppler ultrasound, patient satisfaction questionnaire, TXA
Wied et al. 2015 [182]	TXA (1 g)	Spinal analgesia, plugging of the femoral medullary canal, local analgesia, no drains	Early mobilization, multimodal oral opioid sparing, analgesia, paracetamol, gabapentin, thromboprophylaxis
Wynell-Mayow et al. 2018 [184]	NR	General or spinal anesthesia, multimodal analgesia	Multimodal analgesia
Yu et al. 2018 [189]	Pre-op education	IV TXA 5-10 min before skin incision (20 mg/kg) and 3, 6 h later (1 g) along with 1 g of topical TXA in 50 mL of normal saline solution, no tourniquet or drains, multimodal analgesia	Cold pack on surgical site, dexamethasone, multimodal analgesia, thromboprophylaxis
Zietek et al. 2015 [191]	Pre-op education	Spinal anesthesia, multimodal analgesia, thromboprophylaxis	Early mobilization, multimodal analgesia, cold compression band

Zietek et al. 2016 [192]	NR	Spinal anesthesia, analgesia, cold compression bandages	Early mobilization, multimodal post-operative analgesia
Bernaus et al. 2021 [37]	Anesthetist, blood-saving programme, patient education, antibiotic prophylaxis, oral multimodal analgesia (5 mg diazepam, 20 mg omeprazole, 200 mg celecoxib, 1 g acetaminophen)	Spinal anesthesia (bupivacaine 0.5% 10-12 mg), IV TXA (15 mg/kg), 8 mg dexamethasone, 40 mg omeprazole, LIA (ropivacaine 0.2%, epinephrine 0.5 μ/ml or saline), no drains	Early mobilization, oral multimodal analgesia (celecoxib 200 mg/12 h, acetaminophen 1 g/8 h, sevredol 10 mg)
Berthelsen et al. 2017 [38]	Rehabilitation preparation, case manager, information	NR	NR
Birznieks et al. 2019 [39]	Dexamethasonum 8 mg	Spinal anesthesia (70 mg prilocaine or 18 mg bupivacaine), TXA 1 g, cefazolinum 2 g, infusion (1200 ml crystalloids and 500 ml colloids), LIA 100 ml (ropivacaine 0.75% diluted with 0.9% NaCl), bemiparinum 3500 DV, rivaroxabanum 10 mg	Early rehabilitation, multimodal analgesia (acetaminophen 1 g-500 mg, etoricoxibum 90 mg, morphine 30 mg), TXA
D'Amato et al. 2019 [49]	Check-up with anesthesiologist, epidural chirocaine 0,75% 40 mg, morphine 2 mg, atropine 0,13 mg, PONV prevention	Spinal-epidural anesthesia (chirocaine 0,25% 7 mg)	Tapentadol (100 mg/twice-daily for 4 days) or oxycodone/naloxone (10 mg/5 mg) plus ketoprofen (100 mg/ twice daily), supplemental analgesia (paracetamol 1 g or morphine 0,1 mg/kg)
Dawson-Bowling et al. 2014 [53]	Patients' education and information	Low-dose spinal, plus light general anesthesia (1.5 ml 0.25% bupivacaine, 300 μg diamorphine), drains	Early mobilization, antiemesis, early oral fluid intake, IV cannulae removal, paracetamol, codeine, diclofenac, tramadol, drains removal, RX, Hb check, thromboprophylaxis

De Ladoucette et al. 2020 [54]	Patient information, modern fasting, opioid-sparing multimodal analgesia, high single-dose corticosteroid	Peri-articular injection of local anesthesia (ropivacaine), drain	Early mobilization, thromboprophylaxis, new clinical examination and RX work-up
den Hartog et al. 2017 [59]	Patient education, oral acetaminophen 1000 mg, celecoxib 400 mg, gabapentin 600 mg, IV dexamethasone 0.15 mg/kg, esketamine 15 mg	Spinal anesthesia (low-dose bupivacaine 6-8 mg intrathecally, propofol, esketamine single shot)	Early mobilization, oral acetaminophen 1000 mg, gabapentin 300 mg, tramadol 100 mg, celecoxib 200 mg (for 2 weeks)
den Hartog et al. 2015 [58]	NR	Spinal anesthesia	Early mobilization
Ding et al. 2020 [61]	Patient education, physical therapies, high-protein diet, sleep and fluid management, preemptive analgesia, Hb <70 or 70–100 g/L	General anesthesia, IV fluid (<1500 ml), local infiltration anesthesia, TXA, hypotension control, antibiotic- and thromboprophylaxis, no drain and catheter	Early mobilization, opioid-sparing multimodal analgesia, thrombo- and PONV prophylaxis, anemia management
Dwyer et al. 2012 [64]	Patient education and information, pre-assessment check, calorie and carbohydrate loading, no premedication	Spinal or epidural anesthesia (propofol), drain, no urinary catheter	Early mobilization, non-opiate and non-steroidal analgesia, early oral nutrition, oral fluids and protein and carbohydrate-rich drinks, pneumatic intermittent calf compression, enoxaparin 40 mg, drain removal
Füssenich et al. 2020 [72]	Patient education and information	Thromboprophylaxis, physiotherapy, ergotherapy, pain management, pressure bandage, RX	Early mobilization, pressure bandage removal and ergotherapy, blood examination, medication and pain killers, outpatient consult, RX
Gomez et al. 2019 [75]	NR	General anesthesia and FNB	Early mobilization and nutrition, RX

Gomez et al. 2020 [76]	NR	General anesthesia and FNB	Early mobilization and nutrition, RX
Götz et al. 2021 [77]	Patient education, pre-emptive NSAIDs (etoricoxibe 90 mg 1 h before)	Spinal anesthesia (prilocaine 1% hyperbaric 4 ml = 80 mg and sufentanil 10 µg), IV dexamethasone (8 mg), LIA, subcutaneous 200 mg ropivacaine and 0.5 mg adrenalin, TXA (1 g IV and 2 g topically), no drains	Early mobilization, cardiovascular stimulating and thromboprophylaxis exercises, 3 mg piritramide, analgesia (oral ibuprofen 600 mg 3 times daily, metamizole 500 mg 4 times daily, tramadol 100 mg, oxycodone 10 mg)
Gromov et al. 2015 [78]	NR	General anesthesia	NR
Gupta et al. 2014 [81]	Patient education and information, geriatric and nutritional assessment, thromboprophylaxis, IV fluids, pressure sore prevention, bowel/bladder care and prophylactic antibiotic regimes, oral supplements	NR	Early mobilization and rehabilitation
Hansson et al. 2015 [83]	Inclusion in fast-track system made by ambulance nurse/paramedic when arriving patient	NR	General and local complications evaluation
Hartog et al. 2015 [84]	Oral paracetamol (acetaminophen) 1000 mg, celecoxib 400 mg, omeprazole 20 mg once a day, gabapentin 600 mg, IV dexamethasone 0.15 mg/kg, esketamine 15 mg, PROMs, EQ-5D and NRS	Spinal anesthesia (bupivacaine 6-8 mg)	Oral paracetamol 1000 mg, gabapentin 300 mg, oxycodone (OxyContin) 10 mg, celecoxib (Celebrex) 200 mg, piritramide (Dipidolor) 10 mg intra-muscular

Herndon et al. 2020 [87]	NR	Spinal anesthesia (chloroprocaine 60 mg or bupivacaine 8-15 mg)	Multimodal analgesia protocol (NSAIDs, gabapentin, acetaminophen, narcotics)
Huang et al. 2021 [92]	Health and rehabilitation education, laboratory tests, comorbidity consultations, infection prophylaxis, multimode analgesia	Spinal anesthesia, blood-spearing strategies, no drain, compression bandages	Early mobilization, thromboprophylaxis
Imbelloni et al. 2014 [96]	Pre-anesthetic interview	Spinal anesthesia or 7.5-10 mg, tenoxicam 40 mg and dipyrone 40 mg/kg in 50 mL of Ringer's lactate)	Analgesia, 40 mL bupivacaine 0.25%, tenoxicam 20 mg/12 h, dipyrone 1 g and cefazolin 1 g every 6 h
Kang et al. 2019 [105]	Educational program, oral multimodal analgesia (400 mg celecoxib 1 h before, 200 mg for patients > 70 age)	Opioid-free short-acting spinal anesthesia (mepivacaine), IV TXA (1 g), dexamethasone 8 mg, 40 mg parecoxib sodium, 2 L lactated Ringer's	Early mobilization, opioid-sparing analgesia (celecoxib 200 mg, two times a day), tramadol (100-200 mg up to two times a day), PONV
Klapwijk et al. 2017 [108]	NR	NR	Questionnaires (HOOS-PS, OHS, EQ-5D, SF-12, ICOAP), LMWE (once a day for 4 weeks)
Kolodziej et al. 2020 [110]	IV biofazolin 1-2 g, TXA 15 mg/kg	Spinal epidural anesthesia (Marcaine Spinal 0.5% Heavy-bupivacaine hydrochloride), venous thromboprophylaxis	Early mobilization, thromboprophylaxis, analgesic regimen (ketoprofen 100 mg every 12 h, IV paracetamol 1.0 g every 6 h, oral 50–100 mg tramadol)
Larsson et al. 2016 [116]	RX	NR	NR
Leiss et al. 2021 [117]	Gait training with crutches, multidisciplinary lecture, etoricoxib 90 mg 1 h before	Spinale anesthesia (prilocaine 1% hyperbaric 4 mL = 80 mg, sufentanil 10 µg, dexamethasone 8 mg IV), TXA	Early mobilization, standardized pain management

		(2 g topically and 1 g IV), LIA (200 mg ropivacaine, 0.5 mg adrenalin), no drain	
Li et al. 2020b [120]	Psychological counseling, nutrition and anemia evaluation, fasting and water, PONV prophylaxis	Urinary catheter, drain	Early mobilization, multimodal analgesia (ropivacaine 2.0 mg/ml, IV NSAIDs, flurbiprofen 2 mg/kg, dexibuprofen)
Mikkelsen et al. 2014 [133]	Patient information	Spinal anesthesia, multimodal pain management	Enforced mobilization and nutrition
Okamoto et al. 2016 [138]	NR	Spinal anesthesia	Early mobilization
Otte et al. 2011 [139]	Patient education, pain management (gabapentin 600 mg, slow-release acetaminophen 2 g, celecoxib 400 mg)	Spinal anesthesia (3 ml isobaric bupivacaine 0.5%), IV TXA (1 g) and 1.5 g cefuroxime	Early mobilization, thromboprophylaxis, oral pain management (slow-release 1-2 g/6-12 h acetaminophen, gabapentin 300-600 mg, celecoxib 200 mg/12 h and 5 mg oxycodone on request)
Pollmann et al. 2016 [150]	IV fluids, oxygen, pain relief, ECG, opiate sparing, fluid treatment, transfusion-triggers, thromboprophylaxis	Spinal or general anesthesia	Early mobilization, opiate sparing, fluid treatment, transfusion-triggers, thromboprophylaxis
Porsius et al. 2018 [151]	Patient questionnaires	NR	Early mobilization, pain relief, questionnaires
Specht et al. 2011 [165]	NR	Combined spinal anesthesia and light general anesthesia, catheter	Oral pain treatment, catheter removal, 51 mL LIA injected through the catheter in fast-track with LIA + LINFA group

Talboy et al. 2016 [170]	Oral paracetamol, tramadol	Spinal anesthesia (bupivacaine), no intrathecal opiates, sedation, light general anesthesia, ondansetron, dexamethasone, diclofenac, IV fluid (1000 ml), peri-articular catheter	Analgesic, liquid, PONV
Tan et al. 2018 [171]	Multidisciplinary patient information	Local anesthesia, multimodal analgesia, NSAIDs or COX-2 inhibitor, TXA, no drains and catheters	Early mobilization
Temporiti et al. 2020 [172]	NR	NR	Mobilization and walking the day of surgery or the day after surgery
Vesterby et al. 2017 [179]	Patient information	NR	Early mobilization
Xie et al. 2019 [185]	Doppler ultrasound, comorbidity assessment, oral carbohydrate treatment	General or spinal or combined spinal-epidural anesthesia, IV TXA 15 mg/kg, NSAIDs, physical prophylaxis, chemoprophylaxis, fluid therapy	Early mobilization
Zhang et al. 2020 [190]	Patient information, antibiotics prophylaxis, glucose drink	Local or general anesthesia, catheter, antibiotics	Early mobilization, 50-100 mL warm water, liquid-semi-fluid soft food-normal diet, analgesia
Adams et al. 2021 [26]	Patient education, cardiology and orthopedic consultation, anesthesia evaluation, chlorhexidine washes, mupirocin antibiotic ointment to nares, analgesic regimen (300-600 mg gabapentin, 200 mg celecoxib, 1000 mg acetaminophen)	Spinal or general anesthesia	Analgesic regimen (celecoxib, acetaminophen, tramadol, oxycodone), 81 mg aspirin or 2.5 mg apixaban, thromboprophylaxis, outpatient urological follow-up

Alvis et al. 2021 [28]	Analgesia (acetaminophen, meloxicam, gabapentin)	Analgesia (ropivacaine, epinephrine, ketorolac)	Analgesia (acetaminophen, gabapentin, meloxicam, oxycodone), PONV, fluid administration, rehabilitation
Andreasen et al. 2017 [29]	Information, multidisciplinary symposium	Spinal anesthesia, TXA, LIA, no drains	Early mobilization, opioid-sparing multimodal analgesia, RX
Awada et al. 2019 [32]	Information, cognitive tests, LIA (2 g slow-release acetaminophen, 600 mg ibuprofen, 600 mg gabapentin), 125 mg methylprednisolone	Spinal or general anesthesia	Early mobilization, opioid-sparing multimodal analgesia (acetaminophen 2 g/12 h, ibuprofen 600 mg/12 h, gabapentin 300 mg morning and 600 mg evening), cognitive tests
Berg et al. 2018 [34]	Information	Spinal anesthesia, LIA, TXA, antithrombotic prophylaxis (cloxacillin), no drains	Early mobilization (within 3-6 h after)
Berg et al. 2020 [35]	NR	NR	Early mobilization
Berg et al. 2021 [36]	Oral and written information, admission on the day of surgery	Spinal anesthesia without opioids, multimodal analgesia	Early mobilization
Bjerregaard et al. 2015 [40]	NR	Low-dose spinal or general anesthesia, fluid restrictive, LIA, urinary catheter in fast-track catheterized group	Opioid-sparing multimodal analgesia, early mobilization
Bjerregaard et al. 2016 [41]	NR	Low-dose spinal or general anesthesia, fluid, LIA	Early mobilization, opioid-sparing multimodal analgesia
Castle et al. 2021 [43]	Patient education	Regional adductor canal nerve blocks for KA	Early mobilization, physiotherapy, oral pain medications
Christelis et al. 2015 [45]	Consulting, preadmission review, minimal fasting (clear oral fluids up to 2 h before and carbohydrate	Spinal (not epidural) and local anesthesia (LIA or FNB), IV morphine (≤10 mg), no excessive IV	Early mobilization, multimodal analgesia (oral NSAIDs or COX-2

	loading), no sedative premedication, pre-emptive analgesia (paracetamol, gabapentinoids)	fluids, active warming, antiemetic prophylaxis	inhibitor), early carbohydrate supplementation
Davies et al. 2018 [52]	TXA	Spinal and/or general anesthesia	NR
Drosos et al. 2020 [63]	Preadmission clinic (exercises), blood management program (Hb < 12 gr/dL), antibiotic prophylaxis (cefoxitin sodium), anticoagulation protocol (LMWE)	Spinal or general anesthesia, TXA (2 g in 100 ml normal saline locally applied), transfusion trigger of 9 g/dL	Early mobilization, opioid-sparing analgesia (gabapentinoids), LIA (ropivacaine 1% 3 mg/kg), paracetamol, NSAIDs
Fenelon et al. 2018 [66]	IV TXA and cefuroxime	Spinal anesthesia, enoxaparin and rivaroxaban, tourniquet (for TKA), no drain, two units of blood (if Hb < 12 g/dl)	Early mobilization
Frassanito et al. 2020 [71]	Pre-habilitation programs, information, selective bowel preparation, no premedication, minimal fasting and carbohydrate loading	Spinal or general anesthesia, morphine 100 mcg or sufentanil 2.5–5 mcg or LIA (ropivacaine, ketorolac, epinephrine) or peripheral nerve block (ropivacaine), tourniquet, normothermia, antimicrobial (cefazolin or clindamycin and vancomycin) and antiemetic prophylaxis (dexamethasone 4mg and ondansetron 4mg), analgesia (paracetamol and ketoprofen), TXA (20 mg/kg), PONV, no urinary catheter and drain	Early mobilization, opioid-sparing multimodal analgesia (oral paracetamol 1 g, celecoxib 200 mg, oxycodone 5-10 mg and tramadol 100 mg/ml), early oral fluids and solids intake, RX, blood tests
Galbraith et al. 2017 [73]	Patient education and information, paracetamol, gabapentin, COX-2 inhibitor	Spinal anesthesia, femoral tourniquet, TXA (1 g, 15 min prior), LIA (50 ml 0.2% ropivacaine and 120-150 ml	Early mobilization

		ropivacaine 0.2% + 10 µg/ml epinephrine), no drain	
Glassou et al. 2014 [74]	Information	Spinal anesthesia, LIA, fluid therapy, blood-sparing strategies, no drain, compression bandages, cooling	Early mobilization, thromboprophylaxis, opioid-sparing multimodal analgesia
Halawi et al. 2019 [82]	Preemptive analgesia: 200 mg celecoxib (or meloxicam 15 mg), 5-10 mg oxycodone every 4 h, 975 mg acetaminophen every 8 h	General or combined spinal/general anesthesia, no catheter placement or catheter placement, opioid requirements, local analgesia	Opioid requirements, bladder scan (catheterization if > 350 cc; < 350 cc repetition of bladder scan in 2 h), calculation of the maintenance fluid
Holm et al. 2014 [89]	Multidisciplinary education, oral gabapentin (600 mg), paracetamol (2 g), celecoxib (200 mg) or todolac (200 mg)	Lumbar spinal anesthesia (12.5 mg isobaric bupivacaine 0.5% for THA and 7.5 mg hyperbaric bupivacaine 0.5% for TKA), cefuroxime (1.5 g) and TXA (1 g) 15 min before, LIA (for TKA)	Early mobilization oral morphine 10 mg (if VAS > 50 mm)
Husted et al. 2016 [95]	NR	Spinal anesthesia, TXA, LIA, no drains	Early mobilization, opioid-sparing multimodal analgesia, fixed functional discharged criteria, discharge directly to home
Husted et al. 2012 [94]	NR	NR	NR
Husted et al. 2011b [12]	Multidisciplinary seminar, no extended prophylaxis	Spinal anesthesia (1.5–2.5 mL 0.5% hyperbaric or plain bupivacaine), LIA (0.9 % saline 5 mL/kg/h, colloid Voluven 7.5 mL/kg/h), TXA (1 g)	Transfer to PACU after 1-2 h, encouragement to ambulate upon arrival, physiotherapy, opioid-spearing analgesia (COX-2 inhibitor celecoxib 200 mg/12 h, paracetamol slow release 2 g/12 h, gabapentin 300 mg morning and 600 mg evening)

Jørgensen et al. 2021 [103]	NR	High dose MP (125 mg) with peripheral nerve blocks (at discretion of anaesthesiologist)	Functional discharge criteria
Jørgensen et al. 2017 [102]	Questionnaire on comorbidity	Multimodal analgesic protocol (MP 125 mg)	NR
Jørgensen et al. 2013b [101]	NR	NR	NR
Jørgensen et al. 2013a [24]	NR	Spinal anesthesia, opioid-sparing multimodal analgesia, no peripheral nerve blocks	Early mobilization, functional discharge criteria, discharge to home
Jenny et al. 2020 [97]	Antithrombotic prophylaxis (LMWE or similar molecules for 85% patients, DOACs for 15%)	TXA injection (82% patients)	Early mobilization
Krenk et al. 2014 [113]	Patient information (surgical procedure, analgesia, anesthesia, LOS, physiotherapy), VVLT, concept shifting and stroop color word test, letter digit coding task, screening for sleep apnea and dementia	Spinal anesthesia without opioid, ephedrine 5-10 mg or phenylephrine 0.1 mg (if arterial blood pressure < 25% of baseline, and systolic blood pressure kept > 80-90 mmHg)	Early mobilization, paracetamol, NSAIDs (tramadol, oxycodone, or morphine), VVLT, concept shifting test, stroop color word test, letter digit coding task, questionnaire on comorbidities, daily medication, educational status, sleep pattern
Krenk et al. 2012 [112]	Patient information	Spinal and general anesthesia	Early mobilization, pain regime (paracetamol 1 g every 6 h, gabapentin 300 mg morning and 600 mg evening, tramadol 50 mg every 6 h, celecoxib 200 mg every 12 h, ibuprofen 400 mg every 8 h, morphine 5 mg)
Kort et al. 2018 [111]	Bladder volumes monitoring, oral TXA (1 g if weight < 100 kg, 1.5 g	Spinal or general anesthesia, IV fluid restriction (1000 mL), LIA, IV	Early mobilization, catheter removal (day 1), bladder volume

	if weight > 100 kg), opioid-sparing pain protocol (meloxicam 15 mg, paracetamol 1 g, gabapentine 600 mg, pantoprazole 40 mg)	dexamethasone (8 mg), oral TXA (1 g if weight < 100 kg, 1.5 g if weight > 100 kg)	monitoring, no wound drains, opioid-sparing pain protocol (meloxicam 15 mg, paracetamol 1 g, gabapentine 300 mg and pantoprazole 40 mg)
Kerr et al. 2017 [106]	Pre-assessment	Anesthesia, IV broad spectrum antibiotics	Early mobilization, physiotherapy, IV antibiotics and venous thromboprophylaxis
Lovecchio et al. 2016 [127]	NR	NR	NR
Machin et al. 2013 [128]	Oral gabapentin 300 mg (the night and 2 h before)	Gabapentin 300 mg, 30 mg lansoprazole, 5–10 mg OxyContin, spinal anesthesia and light general anaesthetic, LIA (50 ml 0.25% levobupivacaine, 30 mg ketorolac, 0.5 ml 1:1,000 adrenaline)	20 ml levobupivacaine 0.25%, 0.5 ml 1:1,000 adrenaline diluted up to 40 ml with 0.9% saline (16-20 h after), analgesia (paracetamol, ibuprofen, 5–20 mg OxyContin twice daily for 3 days, OxyNorm and PCA)
Memtsoudis et al. 2020 [132]	Fast-track components: (1) regional anesthesia use; (2) multimodal analgesia use; (3) TXA; (4) anti-emetics use (day 0); (5) steroid use (day 0); (6) physical therapy (day 0 or 1); avoidance (7) wound drains or (8) urinary catheter		
Petersen et al. 2017 [141]	Patient questionnaires	Spinal anesthesia, multimodal opioid-sparing analgesia without peripheral nerve blocks	Early mobilization functional discharge criteria
Petersen et al. 2019 [142]	Patient questionnaires	Local anesthesia, multimodal opioid sparing analgesia	Early mobilization, in-hospital thromboprophylaxis if LOS ≤ 5 days, functional discharge criteria
Petersen et al. 2020a [143]	Patient information and education	Spinal anesthesia, multimodal opioid sparing analgesia	Early mobilization, in hospital thromboprophylaxis if LOS ≤5 days, functional criteria evaluation

Petersen et al. 2021 [145]	NR	Multimodal opioid-sparing analgesia	Early mobilization, thromboprophylaxis if LOS ≤ 5 days, functional discharge criteria evaluation
Pitter et al. 2016 [147]	Self-completed questionnaires, comorbidity evaluation	Neuroaxial anesthesia, opioid-sparing analgesia, NSAIDs	Early mobilization
Plenge et al. 2020 [148]	Assessment in an optimization clinic, multidisciplinary education and full-body antiseptic wash	Peripheral nerve block and/or local infiltration analgesia, antimicrobial prophylaxis, TXA, prevention of perioperative blood loss with anti-fibrinolytics and normothermia	Early mobilization, multimodal opioid-sparing analgesia with paracetamol and NSAIDs on the first 3 days
Robinson et al. 2014 [153]	Pre-op education	Spinal or general, anesthesia, TXA, no drains	Early mobilization, paracetamol 4 times a day with a weak opiate 4 times a day and diclofenac 3 times a day, morphine and weak opiates as required
Romano et al. 2021 [154]	Education and counseling, comorbidities evaluation, minimal pre-op fasting, pre-emptive oral analgesia (oxycodone naloxone)	Selective subarachnoid anesthesia, local analgesia, short-acting sedative hypnotic agents, minimally invasive surgery, TXA, tourniquet, hemostasis, no drains or catheters, restricted fluid balance, hypothermia prevention	Early mobilization, antalgic posture, multimodal opioid sparing analgesia, intermittent cryo-compression, wound management algorithm, negative pressure wound therapy, multimodal PONV prophylaxis, regular diet within 4 h after surgery
Savaridas et al. 2013 [158]	NR	NR	NR
Stambough et al. 2019 [167]	Acetaminophen, celecoxib, oxycontin, midazolam	General anesthesia, no peripheral nerve blocks, dexamethasone and TXA	Analgesic and pain therapy

Starks et al. 2014 [168]	Pre-op education	Spinal anesthesia antibiotic prophylaxis, normothermia maintenance	Early mobilization, analgesic therapy (ovoid opiates when possible)
Stowers et al. 2016 [169]	Patient education	Spinal or epidural anesthesia, +/- morphine antiemetics, TXA and cephazolin within 1 h of incision, catheter avoidance of surgical drains	Early mobilization, analgesia (non-opioid, opioid PCA morphine/oxycodone/fentanyl if required, removed morning of 1st postoperative day), antiemetics and aperients, drains removal
Tucker et al. 2016 [173]	Pre-op warming, carbohydrate drinks, analgesia, analgesic premedication on day of surgery	Spinal anesthesia, capsular LIA, no catheter, TXA 1 g	Early mobilization, early oral hydration, thromboprophylaxis, analgesia
Van Horne et al. 2019a [176]	JointCoach, pre-op education, non-opioid pain medication (acetaminophen, meloxicam, or celecoxib)	Spinal anesthesia, multimodal analgesia, TXA, no drains	Early mobilization, multimodal pain regimen (non-opioid analgesics and a 7-day opioid supply)
Van Horne et al. 2019b [177]	JointCoach, pre-op education, non-opioid pain medication (acetaminophen, meloxicam, or celecoxib)	Spinal anesthesia, multimodal analgesia, TXA, no drains	Early mobilization, multimodal pain regimen (non-opioid analgesics and a 7-day opioid supply)
Winther et al. 2015 [183]	Oral and written information about surgery	Spinal and/or local analgesia, systemic analgesia, multimodal analgesia (paracetamol, dexamethasone, etoricoxib), intra-op fluid, TXA (max. 1.5 g)	Early mobilization, multimodal opioid-sparing analgesia, evaluation pain and at rest and pain at mobilization
Xu et al. 2019 [186]	NR	NR	NR
Yanik et al. 2018 [188]	Pre-op education	Spinal anesthesia, no FNB, standardization of dressings, multimodal pain management	Early mobilization

Abbreviations: TXA = tranexamic acid; GDHM = Goal directed hemodynamic management; PPV = pulse pressure variability; SVV = stroke volume variability; CO = cardiac output; h = hours; LIA = local infiltration analgesia; IV = intravenous; NR = not reported; min = minutes; PONV = postoperative nausea and vomiting; PCA = patient-controlled analgesia; TIVA = Total intravenous anesthesia; LMWE = low-molecular-weight eparin; Hb = hemoglobin; NSAIDs = nonsteroidal anti-inflammatory drugs; MME = Morphine Milligram Equivalents; LOS = hospital length of stay; ESP = erector spinae plane; ACB = adductor canal block; ABT = allogeneic blood transfusions; OA = osteoarthritis; EQ = Euro Quality of Life; PES = pathway management solution; OKS = Oxford Knee Score; CACI = Adjusted Charlson Comorbidity Index; TED = thromboembolic decompression stockings; PACU = post-anesthesiological care unit; MP = methylprednisolone; FNB = femoral nerve block; LB-PAI = liposomal bupivacaine pericapsular injection; ACC = adductor canal catheter; iPACK = posterior capsule single shot block; pre-op = preoperative; IA = intra-articular; post-op = postoperative; PROMS = Patient-reported outcome scores; NRS = numerical rating scale; ICOAP = intermittent and constant osteoarthritis pain score; SF-12 = 12-item Short Form Health Survey; OHS = Oxford hip score; HOOS-PS = hip injury and osteoarthritis outcome score physical function short form; LINFA = local infusion analgesia; COX-2 = cyclooxygenase-2; KA = knee arthroplasty; TKA = total knee arthroplasty; THA = total hip arthroplasty; VAS = visual analog scale; DOACs = direct oral anticoagulants; VVLT = Visual Verbal Learning Test; intra-op = intraoperative.

Table S8. Designation of positive, neutral and negative outcome for each examined study.

Surgery	LOS	Pain	Drain/catheter removal time	Functional recovery	Opioid consumption	Operative time	Costs	Blood loss/ transfusion	Complication/ readmission	Early food recovery	Patient satisfaction	Early mobilization	Discharge
Elbow	N=↓1/2	N=↓1/2	N=↓1/2	N=↑1/2									
Thorax	N=↓2/4	N=↓2/4	N=↓2/4		N=↓2/4	N=↓1/4			N=↓1/4			N=↑1/4	
Spine	N=↓18/25<br>N=1/25 (=)	N=↓8/25	N=↓2/25	N=↑1/25	N=↓4/25	N=↓4/25<br>N=1/25 (=)<br>N=↑1/25	N=↓4/25	N=↓7/25<br>N=1/25 (=)	N=↓6/25	N=↓3/25	N=↑1/25	N=↑2/25	N=↑1/25
Hip/Knee	N=↓47/143	N=↓26/143	N=↓3/143	N=↑15/143	N=↓16/143	N=↓6/143	N=↓6/143	N=↓16/143	N=↓29/143	N=↑1/143	N=↑18/143	N=↑10/143	N=↑12/143

	N=4/143 (=)	N=5/143 (=)<br><br>N=3/143 (↑)	N=1/143 (=)	N=3/143 (=)	N=2/143 (=)	N=↑2/143		N=2/143 (=)<br><br>N=↑3/143	N=3/143 (=)			N=2/143 (=)	

Abbreviations: N = number of studies; ↑ = increase; ↓ = decrease; (=) = no difference.
